# Dna2 removes toxic ssDNA-RPA filaments generated from meiotic recombination-associated DNA synthesis

**DOI:** 10.1093/nar/gkad537

**Published:** 2023-06-23

**Authors:** Binyuan Zhai, Shuxian Zhang, Bo Li, Jiaming Zhang, Xuan Yang, Yingjin Tan, Ying Wang, Taicong Tan, Xiao Yang, Beiyi Chen, Zhongyu Tian, Yanding Cao, Qilai Huang, Jinmin Gao, Shunxin Wang, Liangran Zhang

**Affiliations:** Center for Cell Structure and Function, Shandong Provincial Key Laboratory of Animal Resistance Biology, College of Life Sciences, Shandong Normal University, Jinan, Shandong 250014, China; Center for Reproductive Medicine, Cheeloo College of Medicine, Shandong University, Jinan, Shandong 250012, China; Shandong Provincial Key Laboratory of Animal Cell and Developmental Biology, School of Life Sciences, Shandong University, Qingdao, Shandong 266237, China; Center for Reproductive Medicine, Cheeloo College of Medicine, Shandong University, Jinan, Shandong 250012, China; Center for Reproductive Medicine, Cheeloo College of Medicine, Shandong University, Jinan, Shandong 250012, China; Center for Reproductive Medicine, Cheeloo College of Medicine, Shandong University, Jinan, Shandong 250012, China; Center for Cell Structure and Function, Shandong Provincial Key Laboratory of Animal Resistance Biology, College of Life Sciences, Shandong Normal University, Jinan, Shandong 250014, China; Center for Reproductive Medicine, Cheeloo College of Medicine, Shandong University, Jinan, Shandong 250012, China; Center for Reproductive Medicine, Cheeloo College of Medicine, Shandong University, Jinan, Shandong 250012, China; National Research Center for Assisted Reproductive Technology and Reproductive Genetics, Shandong University, Jinan, Shandong 250012, China; Key Laboratory of Reproductive Endocrinology of Ministry of Education, Jinan, Shandong 250001, China; Shandong Provincial Clinical Research Center for Reproductive Health, Jinan, Shandong 250012, China; Center for Reproductive Medicine, Cheeloo College of Medicine, Shandong University, Jinan, Shandong 250012, China; Advanced Medical Research Institute, Shandong University, Jinan, Shandong 250012, China; Center for Reproductive Medicine, Cheeloo College of Medicine, Shandong University, Jinan, Shandong 250012, China; Advanced Medical Research Institute, Shandong University, Jinan, Shandong 250012, China; Center for Reproductive Medicine, Cheeloo College of Medicine, Shandong University, Jinan, Shandong 250012, China; Shandong Provincial Key Laboratory of Animal Cell and Developmental Biology, School of Life Sciences, Shandong University, Qingdao, Shandong 266237, China; Center for Cell Structure and Function, Shandong Provincial Key Laboratory of Animal Resistance Biology, College of Life Sciences, Shandong Normal University, Jinan, Shandong 250014, China; Center for Reproductive Medicine, Cheeloo College of Medicine, Shandong University, Jinan, Shandong 250012, China; National Research Center for Assisted Reproductive Technology and Reproductive Genetics, Shandong University, Jinan, Shandong 250012, China; Key Laboratory of Reproductive Endocrinology of Ministry of Education, Jinan, Shandong 250001, China; Shandong Provincial Clinical Research Center for Reproductive Health, Jinan, Shandong 250012, China; Center for Cell Structure and Function, Shandong Provincial Key Laboratory of Animal Resistance Biology, College of Life Sciences, Shandong Normal University, Jinan, Shandong 250014, China; Advanced Medical Research Institute, Shandong University, Jinan, Shandong 250012, China

## Abstract

During the repair of DNA double-strand breaks (DSBs), *de novo* synthesized DNA strands can displace the parental strand to generate single-strand DNAs (ssDNAs). Many programmed DSBs and thus many ssDNAs occur during meiosis. However, it is unclear how these ssDNAs are removed for the complete repair of meiotic DSBs. Here, we show that meiosis-specific depletion of Dna2 (*dna2-md*) results in an abundant accumulation of RPA and an expansion of RPA from DSBs to broader regions in *Saccharomyces cerevisiae*. As a result, DSB repair is defective and spores are inviable, although the levels of crossovers/non-crossovers seem to be unaffected. Furthermore, Dna2 induction at pachytene is highly effective in removing accumulated RPA and restoring spore viability. Moreover, the depletion of Pif1, an activator of polymerase δ required for meiotic recombination-associated DNA synthesis, and Pif1 inhibitor Mlh2 decreases and increases RPA accumulation in *dna2-md*, respectively. In addition, blocking DNA synthesis during meiotic recombination dramatically decreases RPA accumulation in *dna2-md*. Together, our findings show that meiotic DSB repair requires Dna2 to remove ssDNA-RPA filaments generated from meiotic recombination-associated DNA synthesis. Additionally, we showed that Dna2 also regulates DSB-independent RPA distribution.

## Graphical Abstract

**Graphical Abstract:**
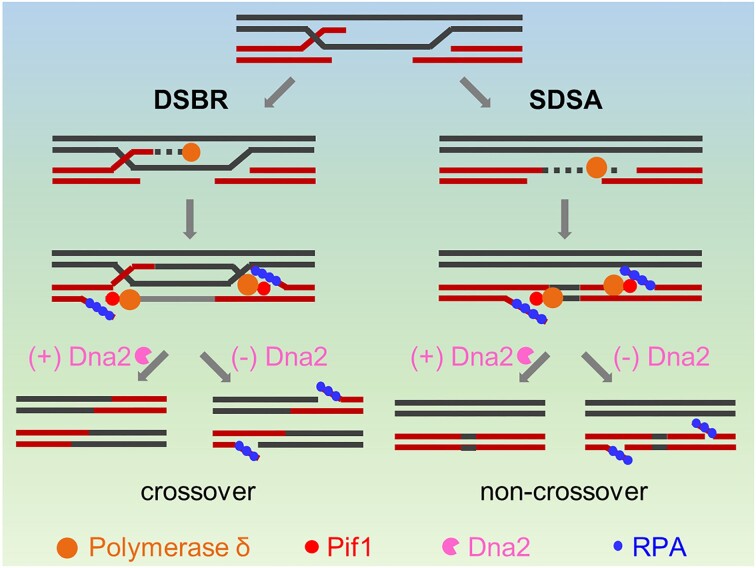

## INTRODUCTION

Meiosis is a specialized cellular program to produce haploid gametes. During meiosis, one round of DNA replication is followed by two successive rounds of chromosome segregation: homologous chromosomes (homologs) segregation in meiosis I, and sister chromatids segregation in meiosis II. The hallmark of meiosis is using the homologs as templates to repair the programmed DNA double-strand breaks (DSBs) and generate crossovers (1,2).

The topoisomerase-like Spo11 works with cofactors to catalyze the formation of DSBs to initiate meiotic recombination (3–5). DSBs are then immediately resected from their 5′-terminal strands to produce 3′ single-stranded tails, which are coated by RPA and later substituted by the RecA family proteins, Rad51 and Dmc1, to form nucleoprotein filaments (6–9). The nucleoprotein filaments mediate DNA strand invasion, which results in the formation of displacement loop (D-loop) intermediates (1,2,10). Subsequently, *de novo* DNA synthesis begins from the 3′ end of the invaded strand. If this invasion strand is displaced and then anneals with the other resected end, the DSB is finally repaired to be a non-crossover (NCO) via the synthesis-dependent strand annealing pathway (SDSA) (11). If this invasion strand is stabilized, the DSB would be repaired via the double-strand break repair (DSBR) pathway. The second end would be captured during this process to form a double Holliday junction (dHJ), which would be resolved as a crossover (CO) (12) (Supplementary Figure S1).

Both SDSA and DSBR pathways require *de novo* DNA synthesis from the invasion end and also the other end to fill the gap (13). Extensive studies show that DNA polymerase δ (Pol δ) catalyzes *de novo* DNA synthesis in both mitotic and meiotic nuclei (14–17). The newly synthesized DNA could displace the parental strand to generate single-strand DNA (ssDNA) flaps via the strand displacement activity of Pol δ (16,17). These ssDNA flaps could be coated by RPA and visualized as ssDNA-RPA filaments. The simple ssDNA and ssDNA-RPA filaments need to be efficiently and timely removed to restore genome integrity. For meiotic nuclei, numerous programmed DSBs have the potential to generate a considerable number of ssDNA-RPA filaments. Thus, the timely removal of these filaments is a big challenge. However, the molecules involved in removing these ssDNA-RPA filaments are still unknown (Supplementary Figure S1).

During the lagging-strand DNA replication, each Okazaki fragment is initiated by the formation of a primer composed of RNA and initiator DNA. When Pol δ encounters the 5′-end of a preceding Okazaki fragment, this fragment may be dissociated from the template strand by Pol δ through its strand displacement activity (18,19). Extensive studies show that Rad27 (Fen1) is the leading nuclease removing a majority of 5′-ssDNA flaps in *S. cerevisiae* (20,21). The rest, a small portion of 5′-ssDNA flaps that are not timely removed by Rad27, are extended to form long ssDNA flaps by continuous DNA synthesis and are coated by RPA. *In vitro* studies show that ssDNA-RPA flaps are suitable substrates for Dna2 helicase/nuclease (22,23). Therefore, Dna2 probably provides the final safeguard in removing the ssDNA flaps during the lagging strand DNA synthesis (19,22,24). One recent study directly analyzing Okazaki fragments synthesized *in vivo* found that Rad27 processes the majority of the 5′-ssDNA flaps of lagging strands and Exo1 makes a significant additional contribution, however, the role of Dna2 in this process is extremely limited (25). Therefore, the role of Dna2 in removing long ssDNA flaps generated in DNA replication is still not so clear.

Here, we systematically explored the role of Dna2 in meiosis. The meiosis-specific depletion of Dna2 (*dna2-md*) resulted in the accumulation of RPA as well as a high frequency of chromosome mis-segregation and thus inviable spores. The result of ChIP-seq revealed that RPA was enriched at DSBs, probably at resected single DSB ends, in both wild type (WT) and *dna2-md* in a Spo11-dependent manner. Along with the progression of meiotic recombination, RPA enrichment gradually diminished in WT while RPA expanded to broader regions around DSBs in *dna2-md*. As a result, DNA integrity is compromised, however, unexpectedly, the levels of crossovers and non-crossovers seem to be unaffected in *dna2-md*. Pachytene induced expression of Dna2 could efficiently remove the accumulated RPA and rescue spore viability in *dna2-md*. Moreover, the removal of Pol δ activator Pif1 and Pif1 inhibitor Mlh2 in *dna2-md* prevented and stimulated RPA accumulation, respectively. In addition, blocking new DNA synthesis during meiotic recombination by adding hydroxyurea (HU) resulted in a dramatic decrease in RPA accumulation in *dna2-md*. These results support the proposal that Dna2 is required to remove ssDNA-RPA filaments generated during meiotic recombination-associated DNA synthesis. Additionally, Dna2 regulates RPA distribution in a Spo11-independent manner.

## MATERIALS AND METHODS

### Yeast strains

The *Saccharomyces cerevisiae* strains used in this study are derivatives of the SK1 background and listed in Supplementary Table S1. The p*CLB2-DNA2* strain was constructed by replacing its native promoter with the *CLB2* promoter using the polymerase chain reaction (PCR) method (26–29). Similarly, the *pCLB2-PIF1* strain was also constructed by the PCR method. The p*GAL1-DNA2* strain was constructed by replacing the native promoter of the *DNA2* gene with the *GAL1* promoter. The *pSCC1-CDC6* strain was constructed by replacing its native promoter with the *SCC1* promoter using the PCR method (27–30). Site-directed mutagenesis of DNA2E675A and DNA2R1253Q was introduced by PCR. The *DNA2-AID* strain was constructed by inserting an AID (auxin inducible degron) tag into the C-terminal of DNA2. The AID tag was amplified by PCR from a pNAT-AID^71-114^-6HA plasmid (31,32). The DNA2-3HA strain was constructed by inserting a triple-HA epitope into the C-terminal of DNA2 via the PCR method (27–29). The triple-HA tag was obtained from a pKT221 plasmid (33).

### Meiotic time course

The meiotic time course was performed as previously described (34,35). Briefly, yeast cells were patched onto YPG plates (3% glycerol, 1% yeast extract, 2% Bacto peptone, and 2% Bacto agar) and incubated for 14 h at 30°C. Then cells were steaked onto YPD plates (1% yeast extract, 2% Bacto peptone, 2% glucose, and 2% Bacto agar) and incubated for 56 h at 30°C. A single healthy colony was inoculated into 4 ml of YPD liquid medium and incubated at 30°C for 24 h with shaking. An appropriate amount of culture was transferred into the SPS medium (0.5% yeast extract, 1% Bacto peptone, 0.67% yeast nitrogen base without amino acids, 1% potassium acetate and 50 mM potassium biphthalate, pH 5.5) and cultured at 30°C for 16 h with shaking. Synchronized cells were harvested and transferred into the sporulation medium (SPM; 1% potassium acetate, 0.02% raffinose) to induce meiosis. DAPI staining was used to analyze meiotic nuclear divisions under a microscope. To induce *pGAL1-DNA2* expression, 1 μM β-estradiol was added at the desired time points during meiosis. To induce the degradation of Dna2, 25 μM CuSO_4_ and 2 mM auxin (3-indoleacetic acid) (or DMSO as a control) were added to the cultures of DNA2-AID at desired time points.

### Sporulation efficiency and spore viability assays

After the cells were transferred to SPM liquid medium for 24 h, sporulation efficiency was analyzed by examining the frequency of cells with asci under a light microscope. Tetrads were dissected onto YPD plates under a dissection microscope and incubated at 30°C for 3 days. Spore viability was determined as the frequency of viable spores over the expected total spores (the number of dissected tetrads times 4).

### Spore-specific fluorescence assay of chromosome mis-segregation

The spore-specific promoter-driven YFP (*P^YKL050C^-YFP*) and RFP (*P^YKL050C^-RFP*) were separately integrated into the allelic position near the centromere of chromosome 9 for the two parental haploids (36). Yeast cells were patched onto YPD plates and incubated at 30°C for 14 h. Then the fresh cells were transferred into 4 ml of SPM and cultured at 30°C for 2–3 days with shaking. Spore patterns with distinct fluorescence in tetrads were determined using a Zeiss fluorescence microscope (AxioImager.Z2). The four spores in one tetrad with accurate chromosome segregation produce two spores expressing YFP and the other two spores expressing RFP, while tetrads with mis-segregated chromosome 9 exhibit abnormal patterns of yellow/red fluorescence. The chromosome 9 mis-segregation frequency was calculated as the number of tetrads with mis-segregated chromosome 9 divided by the total number of tetrads with YFP/RFP spores.

### Chromosome spreading and immunofluorescence

For chromosome spreading, synchronized yeast cells were collected at appropriate time points, processed into spheroplasts with Zymolyase 100T, spread on a clean glass slide with 1% Lipsol, and fixed using 3% paraformaldehyde containing 3.4% sucrose (37). For immunostaining, slides with spread nuclei were first dipped into 0.2% Photo-Flo for 30 seconds, then transferred to Tris-Buffered Saline (TBS) (136 mM NaCl, 3 mM KCl and 25 mM Tris–HCl, pH 8.0), and subsequently incubated at room temperature for 15 min. The slides were blocked with 1× TBS with 1% BSA for 10 min and incubated with the appropriate primary (4°C overnight) and secondary (37°C for 2 h) antibodies. The primary antibodies used in this study included rabbit polyclonal anti-RPA (Agrisera, Cat# AS07214), rat monoclonal anti-HA (Roche, Cat#11867423001), goat polyclonal anti-Zip1 (Santa Cruz Biotechnology, Cat# sc-48716) and mouse monoclonal anti-Myc (Santa Cruz Biotechnology, Cat# sc-40). The secondary antibodies used in this study were Alexa 488-conjugated donkey anti-mouse/goat/rabbit (Thermo Fisher Scientific, Cat#A-21202/A-11055/A-21206) and Alexa 594-conjugated donkey anti-rat/goat (Thermo Fisher Scientific, Cat# A-21209/A-11058). Chromosomal DNA was stained with DAPI. Stained samples were visualized and imaged under a Zeiss fluorescence microscope (AxioImager.Z2) with an EMCCD camera and appropriate filters.

### Quantification of RPA foci and immunofluorescence intensity

About 100 properly spread nuclei were analyzed at each time point using ImageJ (https://imagej.nih.gov/ij), spread nuclei that were overstretched or only partially spread were excluded in this analysis (38). The following criteria were applied to determine an RPA focus (similarly for a focus of other proteins): (i) located within DAPI stained nuclear area, (ii) were punctate in appearance (and thus well above the background) and (iii) were separated from adjacent foci by at least two pixels (∼0.16 μm; otherwise it was counted as a single focus). The focus number was manually determined with the help of the ‘multi-point’ tool in ImageJ. Samples were counted blindly by two observers. Quantification of RPA immunofluorescence intensity was performed as previously described (34). To accurately measure the intensity of RPA in different cells, the same buffers, the same concentration of antibodies, and the same incubation time were used to immunostaining RPA. The same parameters (e.g. exposure time and laser power) were used for image acquisition. The fluorescence intensity of RPA was quantified with ImageJ software. The target cell was circled according to the DAPI signal and the fluorescence intensity was measured as the raw intensity. The same circle was drawn in a region nearby this cell and the intensity was measured as the background intensity. The RPA fluorescence intensity was calculated as the raw intensity minus the background intensity.

### Western blot

Synchronized yeast cells were collected at appropriate time points in SPM and lysed in 20% trichloroacetic acid (TCA) using glass beads. After centrifuged at 12000 g for 1 min, the resultant pellet was extracted with Laemmli buffer and denatured in boiling water for 5 min. The proteins were separated on 10% SDS-PAGE and transferred to nitrocellulose filter membranes (Millipore, Cat# HATF00010). Primary antibodies used were mouse monoclonal anti-HA (Sigma, Cat# H9658), mouse monoclonal anti-PGK1 (Abcam, Cat# ab113687), rabbit polyclonal anti-Rad53 (Abcam, Cat# ab104232), guinea pig anti-Red1 polyclonal antibody against amino acids 492–709 of budding yeast Red1 protein was prepared by Dai-an Biological Technology Incorporation (Wuhan, China). The quantification of signal intensity was performed using ImageJ software.

### DNA physical assay

DSBs, dHJs, crossovers, and non-crossovers were analyzed by DNA physical assays in combination with Southern blot as described previously (39–41). Briefly, the genomic DNA was extracted from synchronized yeast cells at desired time points in SPM and digested with Xho I. One-dimension (1D) gel electrophoresis was carried out, followed by a Southern blot to detect DSBs, crossovers, and non-crossovers. Two-dimension (2D) gel electrophoresis in combination with Southern blot was performed to detect IH-dHJs and IS-dHJs. For both crossovers and non-crossovers analysis, the genomic DNA was digested with Xho I and NgoM IV, separated by 1D gel electrophoresis, and detected by Southern blot. The probe for the Southern blot was labeled with α-^32^P-dCTP by a random labeling system (Thermo Fisher, Cat#18187-013). All DNA species in Southern blot were visualized using a phosphorimager (the Cyclone Plus Storage Phosphor System, PerkinElmer) and quantified using Quantity One software (Bio-rad).

### Native-denaturing 2D gel electrophoresis

The length of DSB end resection was analyzed by native-denaturing 2D gel electrophoresis followed by the Southern blot as described previously (40,42). Briefly, the genomic DNA of synchronized yeast cells was isolated and digested with Xho I. Subsequently, the DNA was separated on a 0.6% Seakem LE agarose gel in 1 x TBE for 24 h at room temperature for the first-dimension gel electrophoresis, and then the gel was stained with ethidium bromide for 30 min at room temperature. Then the gel slices containing targeted DNA were excised and used for the second-dimension denaturing gel electrophoresis with 1× alkaline running buffer (50 mM NaOH, 1 mM EDTA) for 30 h in a cold room. The DNA species were detected by Southern blot and visualized using a phosphorimager (the Cyclone Plus Storage Phosphor System, PerkinElmer).

### Pulse field gel electrophoresis (PFGE) analysis

The synchronized yeast cells were collected at appropriate time points in SPM, and then the genomic DNA was prepared in plugs of low-melting agarose to avoid shearing as previously described (43,44). For PFGE, chromosomes were separated with a CHEF-DRIII PFGE system (Bio-Rad) with the following conditions: 1% agarose in 0.5× TBE; 15.1 s initial switch time; 25.1 s final switch time; switch angle 120°; 6 V/cm; 14°C; running time 28 h. DNA was detected by Southern blot and signals were visualized using a phosphorimager (the Cyclone Plus Storage Phosphor System, PerkinElmer) and quantified by ImageJ software (https://imagej.nih.gov/ij).

### Yeast alkaline comet assay

The yeast alkaline comet assay was carried out as previously described with minor modifications (45). Synchronized yeast cells were collected and processed into spheroplasts by Zymolyase 100T in S buffer (1 M sorbitol, 25 mM KH_2_PO_4_, pH 6.5). The spheroplasts were collected by centrifugation and washed twice with ice-cold S buffer. The resultant pellets were resuspended in 1.5% (wt/vol) low-melting agarose and spread over a slide coated with 0.5% (wt/vol) normal-melting agarose. The slides were solidified in a cold room. Subsequently, the slides were submerged in lysis buffer (30 mM NaOH, 1 M NaCl, 0.05% laurylsarcosine, 50 mM EDTA, and 10 mM Tris–HCl, pH 10.0) for 20 min at 4°C and then in electrophoresis buffer (30 mM NaOH, 10 mM EDTA, and 10 mM Tris–HCl, pH 10.0) for 20 min at 4°C. The samples were electrophoresed using the same electrophoresis buffer for 8 min at 0.5 V/cm at 4°C. Subsequently, the slides were incubated in neutralization buffer (10 mM Tris–HCl, pH 7.4) for 10 min, 76% ethanol for 5 min, and 96% ethanol for 5 min at room temperature. Samples were stained with DAPI and images were acquired using a Zeiss fluorescence microscope (AxioImager.Z2) with an EMCCD camera.

### Flow cytometry

One hundred microliters of synchronized cells were collected and fixed in 1 ml of 60% ethanol with 0.1 M sorbitol. The fixed cells were washed twice with 0.5 ml of 50 mM Tris–HCl (pH 7.5) and resuspended in 0.5 ml of 50 mM Tris–HCl with 0.1% Tween 20 (Sigma, Cat#P9416), 0.1 mg/ml RNase A (Sigma, Cat#R6513), and 0.5 μl of SYTOX™ Green dead cell stain (Thermo Fisher, Cat#S34860). The cells were incubated at 25°C for 12 h in a dark room with 150 rpm shaking. For each sample, 20 000 cells were sorted by BD LSRFortessa and the result was analyzed by FlowJo (Version 10.8.1).

### ChIP-seq analysis

For chromatin immunoprecipitation of RPA, 10 ml of synchronized yeast cells were crosslinked with 1% formaldehyde for 90 min, and the reaction was quenched with glycine. Cells were collected by centrifugation and washed twice with ice-cold 1× TBS. Then the cells were broken with glass beads in lysis buffer (0.1% SDS, 4 mM EDTA, 20 mM Tris–HCl, pH 8.0), the glass beads and debris were removed by centrifugation, and the chromatin-containing suspension was sheared by sonication. After centrifugation, the supernatant was transferred into a pre-incubated mix containing 2 μl of RPA antibody (AS07-214, Agrisera) and Protein A + G magnetic beads (or Protein A + G magnetic beads only as a control) in a blocking buffer (0.5% BSA, 2 mM EDTA, 150 mM NaCl, 20 mM Tris–HCl pH 8.0, 1% Triton X-100) for 10 h. The beads were washed with buffer I (2 mM EDTA, 20 mM Tris–HCl, pH 8.0, 0.1% SDS, 1% Triton X-100, 150 mM NaCl), buffer II (20 mM Tris–HCl, pH 8.0, 0.1% SDS, 1% Triton X-100, 500 mM NaCl), buffer III (1 mM EDTA, 10 mM Tris–HCl, pH 8.0, 250 mM LiCl, 1% Deoxycholate, 1% NP-40), and buffer IV (1 mM EDTA, 10 mM Tris–HCl, pH 8.0). After being washed, samples were eluted with extraction buffer (1% SDS, 1 mM EDTA, 10 mM Tris–HCl pH 8.0). Eluted samples were reverse crosslinked and DNA was recovered with a QIAquick PCR Purification kit (QIAGEN, Cat# 28106). The library was prepared using the VAHTS Universal DNA Library Prep Kit for Illumina V3 (Vazyme, Cat#ND607-01) according to the manufacturer's manual. The library DNA was sequenced by GENEWIZ using an Illumina HiSeq 2500 instrument with a 2 × 150 paired-end (PE) configuration. Spo11-oligo density was obtained from ref. (46).

### Processing of illumina sequence data

The raw reads were filtered based on the quality value (q25) by Trim-galore (version 0.6.6). BOWTIE2 (Version 2.4.2 (47)) was used to align the filtered reads to the *S. cerevisiae* reference genome (SacCer3) and generate BAM files. To analyze the RPA distribution, the reads of BAM files were normalized to counts per million (CPM) by using DEEPTOOLS (Version 2.30.0 (48)). The enrichment of RPA reads around Spo11-oligo hotspots was detected by computeMatrix (Version 3.5.0 (48)). To analyze the correlation between RPA reads and the counts of Spo11-oligo, the genome was divided into 1kb/bin by MultiBam Summary (version 3.5.0 (48)) and then the Pearson correlation analysis was performed. MACS2 (Version 2.2.7.1 (49)) was used for peak calling with the following parameters: Nomodel mode, 200 bp extension size, the cutoff of FDR (q-value) at 0.05.

### Quantification and statistical analysis

**Figure 1.**
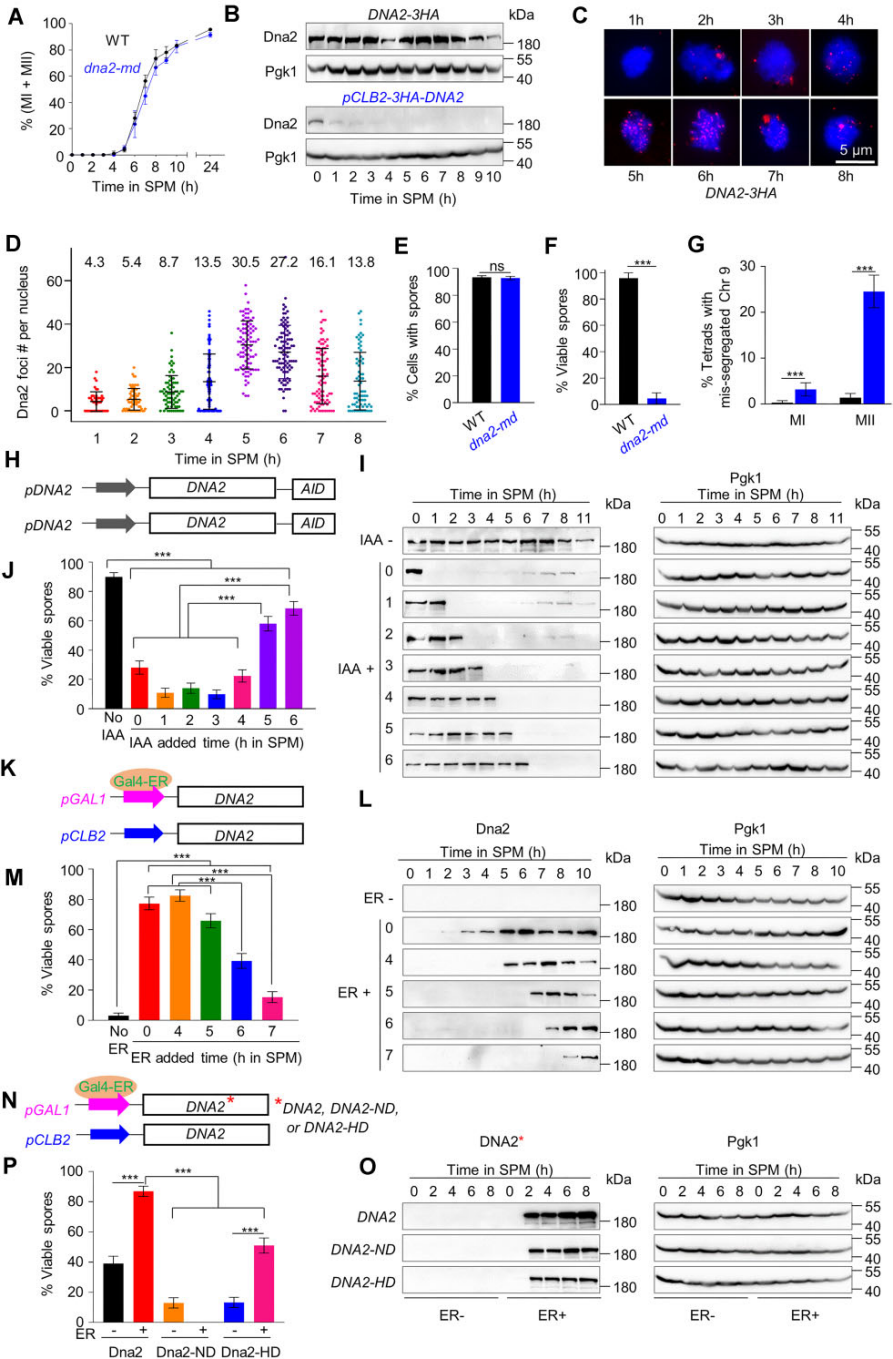
Dna2 is required for normal meiosis. (**A**) Meiotic divisions in WT and meiosis-specific depletion of *dna2* (*dna2-md*). More than 200 nuclei were examined at each time point in each experiment. MI + MII are nuclei that completed at least the first meiotic divisions detected by DAPI staining. (**B**) Western blot to show Dna2 abundance during meiosis. Dna2 was tagged by 3HA and detected by an antibody against HA. (**C**) Representative images to show the dynamics of Dna2 foci in WT during meiosis. (**D**) Quantification of Dna2 foci in (C). Sample size, *n* = 43, 61, 72, 84, 89, 94, 87 and 73 nuclei, respectively. (**E**) Sporulation efficiency. More than 200 cells were examined at 24 h in SPM. (**F**) Spore viability. For each strain, 96 tetrads were analyzed. (**G**) The frequency of tetrads with mis-segregated chromosome 9. 647 (WT) and 566 (*dna2-md*) tetrads were examined. (**H**) Cartoon to show Dna2 tagged with the 3HA-AID at its C-terminal. (**I**) Dna2 abundance examined by western blot. (**J**) Spore viability. For each sample, 96 tetrads were examined. (**K**) Cartoon to show the *pGAL1-DNA2* strain (with 3HA tagged at its N-terminal). (**L**) Dna2 abundance examined by western blot. (**M**) Spore viability. For each sample, 96 tetrads were examined. (**N**) Cartoon to show the *dna2-md* strain with *pGAL1-DNA2*, *pGAL1-DNA2-ND* or *pGAL1-DNA2-HD*. ND: nuclease dead; HD: helicase dead. DNA2, DNA2-ND and DNA2-HD were tagged by 3HA at their N-terminal. (**O**) Dna2 abundance examined by western blot. One micromole β-estradiol (ER) was added at 0h in SPM. (**P**) Spore viability. For each strain, 96 tetrads were analyzed. Error bar, SEM of three independent experiments (A), SD (D) or 95% confidence interval (E, F, G, J, M and P). Scale bar, 5 μm. Two proportion Z-test (E, F, G, J, M and P); ns (not significant), *** (*P* < 0.001).

**Figure 2.**
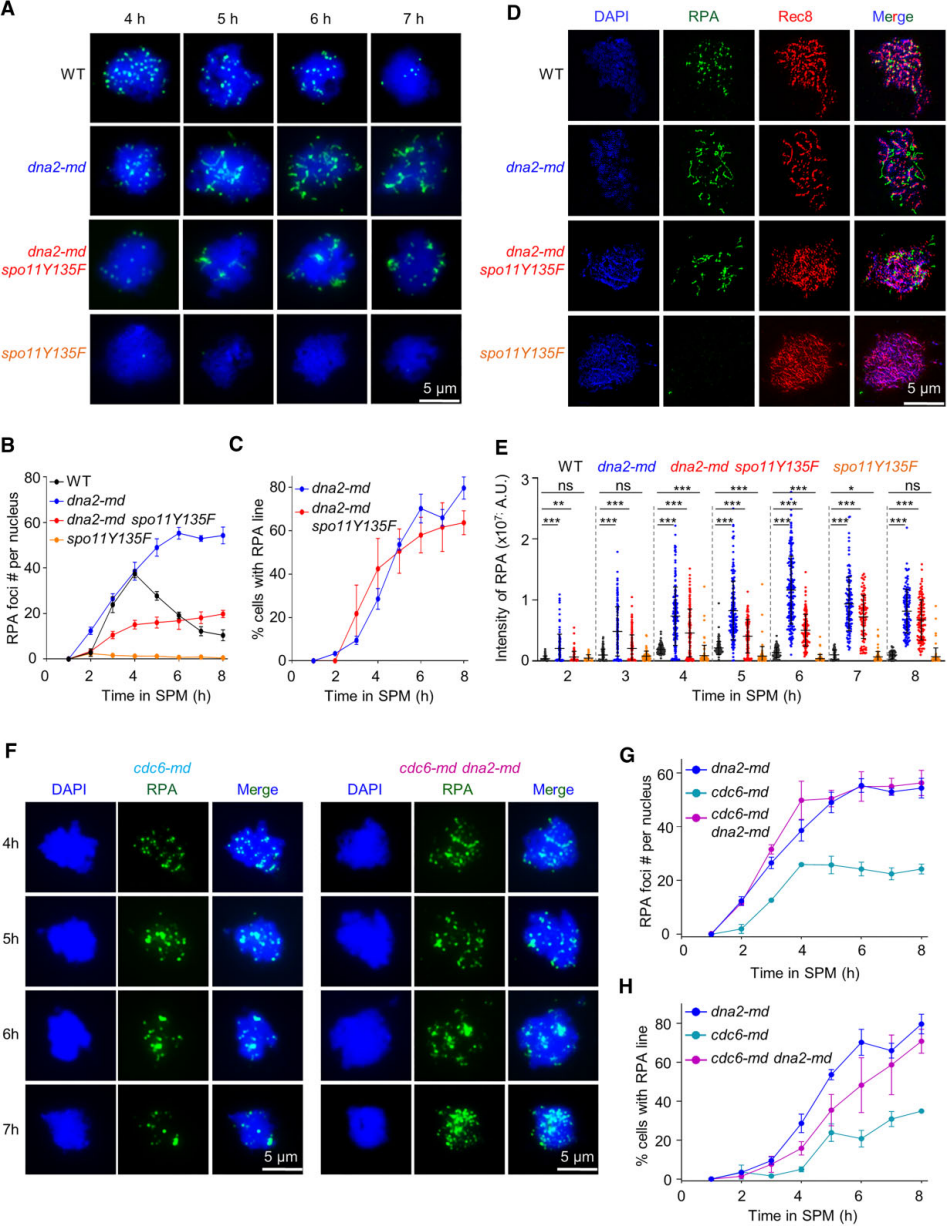
Abundant RPA accumulate in *dna2-md* through Spo11-dependent and -independent manners. (**A**) Representative images to show RPA accumulation. Scale bar, 5 μm. (B, C) Quantification of (A) to show the number of RPA foci (**B**) and the percentage of cells with RPA lines (**C**). Error bar, SEM; *n* = 3. At each time point in each experiment, ∼100 nuclei were examined in each strain. (**D**) Representative images to show RPA examined under a structured illumination microscope (SIM). Cells were collected at 6 h in SPM. (**E**) Quantification of (A) to show per-nucleus RPA intensity. Error bar, SD. Sample size, *n* = 120, 129, 118, 28, 147, 149, 137, 81, 131, 165, 133, 88, 134, 157, 119, 97, 112, 193, 131, 61, 54, 127, 93, 78, 52, 124, 115 and 72 nuclei, respectively. Two-tailed Student's *t*-test; ns (not significant), * (*P* < 0.05), ** (*P* < 0.01) and *** (*P* < 0.001). (**F**) Representative images to show RPA in *cdc6-md* and *cdc6-md dna2-md*. (**G**, **H**) Quantification of (F) to show the number of RPA foci (G) and the percentage of cells with RPA lines (H). Error bar, SEM from three experiments, or the range of two experiments for *cdc6-md*. At each time point in each experiment, ∼100 nuclei were analyzed for each strain. The *dna2-md* data were duplicated from (B and C). Scale bar, 5 μm.

**Figure 3.**
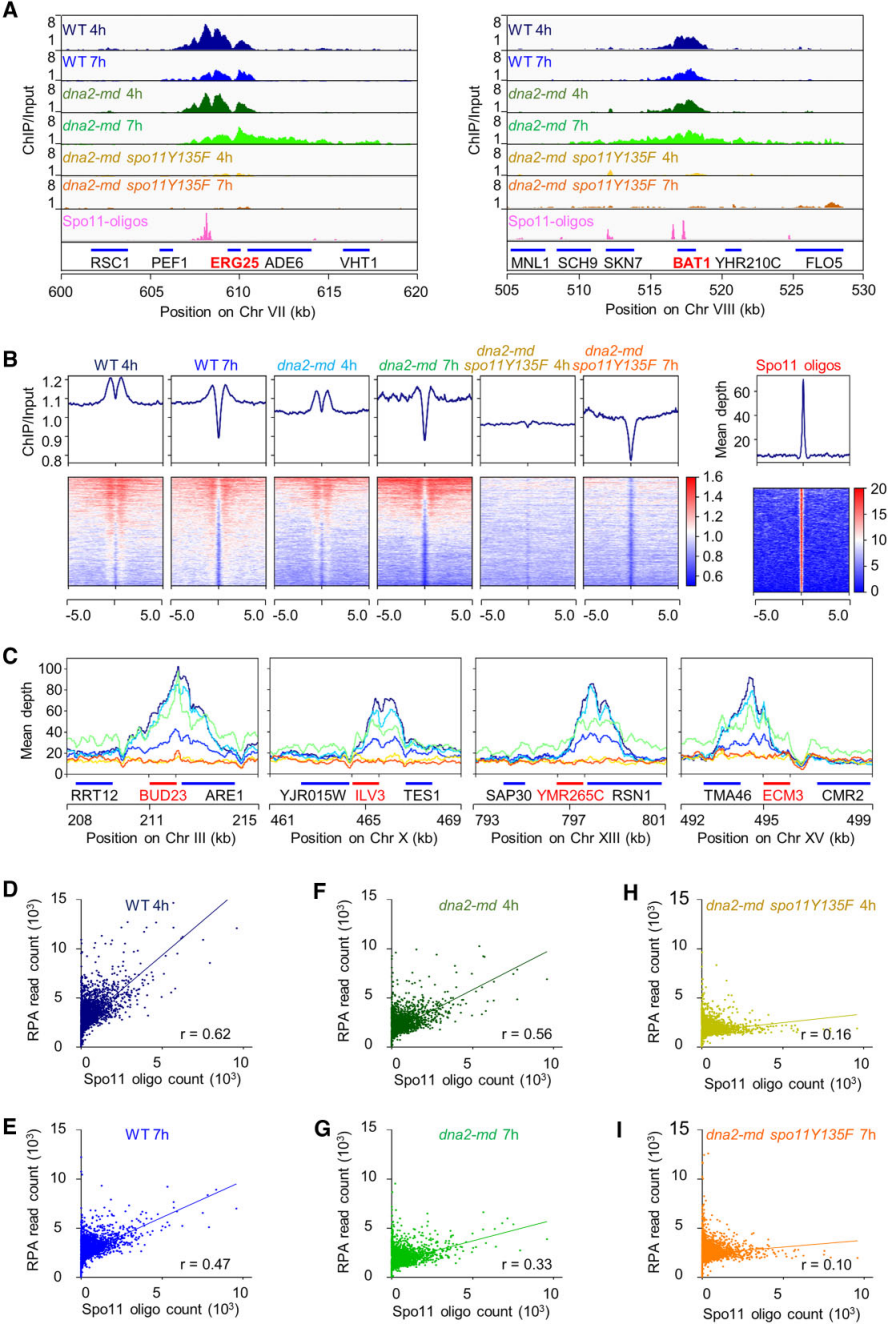
Accumulated RPA expands from DSBs to broader regions in *dna2-md*. (**A**) Snapshots of RPA enrichment at two DSB hotspots, *ERG25* (left panel) and *BAT1* (right panel). (**B**) The heatmap presentation of RPA ChIP-seq signal centered at DSB hotspots. DSB hotspots were identified by Spo11 oligos (46). (**C**) RPA ChIP-seq reads at four DSB hotspots, *BUD23*, *ILV3*, *YMR265C* and *ECM3*. (**D–I**) Correlations between RPA read counts and Spo11 oligo counts in WT (D and E), *dna2-md* (F and G) and *dna2-md spo11Y135F* (H and I). *r*, Pearson's correlation coefficient. RPA read count and Spo11 oligo count were presented as the sum of 1 kb bins.

Data in Figures 1D, 2E, 6B, D, 7B, D, F, Supplementary Figures S6D, and S10C are presented as means ± SD; data in Figures 1E, F, G, J, M, P, 6E, 7C, Supplementary Figures S7C, S8B and C are presented as means ± 95% confidence interval; data in Supplementary Figure S10D are presented as the range of two experiments; other data are presented as means ± SEM. Sample sizes are described in figure legends. GraphPad Prism 8.0 is used for the Pearson correlation analysis in Figure 3D–I. The levels of statistical significance are indicated in figures and figure legends: ns (not significant), *P* ≥ 0.05; *P < 0.05; **P < 0.01; ***P < 0.001.

## RESULTS

### Dna2 is essential for normal meiosis

Dna2 is a highly conserved nuclease and helicase in eukaryotic cells and plays multiple roles in DNA metabolism during mitotic growth, including DNA replication, DSB end resection, stalled replication fork processing, and mitochondrial genome integrity maintenance (50–53). However, the function of Dna2 in meiotic cells is unknown. First, the expression of Dna2 during meiosis was examined. For this purpose, the endogenous Dna2 was tagged with a 3× HA epitope at its C-terminus. For WT, in a well-synchronized meiotic culture in the sporulation medium (SPM), cells go through each step of meiosis at fixed time points (Figure 1A) (34,39,40,54). According to previous reports, most cells complete pre-meiotic DNA replication and undergo DSBs at ∼3 h in SPM (leptotene) in a standard meiotic time-course (39,55). Accompanied by DSB repair, the synaptonemal complex (SC) begins to assemble between homologs and cells gradually reach zygotene at ∼4 h in SPM (39,55). When SC extends to the full homolog axes at ∼5 h in SPM, cells enter pachytene. Most, if not all, DSBs are repaired, and homologous recombination is completed during pachytene (2,40,41). Afterward, SC begins to disassemble from chromosomes and nuclei undergo the first division to segregate homologs at ∼6h in SPM (39,55). Western blot with synchronized meiotic samples showed that Dna2 was constitutively expressed during meiosis (Figure 1B, top). Subsequently, we analyzed the dynamics of Dna2 foci in WT through chromosome spread and immunofluorescence staining (Figure 1C, D). The results showed that the number of Dna2 foci significantly increased at SPM 5 h and was maintained at a high level at SPM 6h, then dramatically decreased, implying that Dna2 is chromosome bound around pachytene.

To further investigate the functions of Dna2 in meiosis, we replaced its native promoter with the *CLB2* promoter, which is active only in mitosis but not in meiosis (26,31). This meiosis-specific depletion mutant, *pCLB2-HA3-DNA2*, was simply named *dna2-md*. In *dna2-md*, Dna2 protein was quickly depleted to an almost undetectable level after 1h upon meiosis induction in SPM (Figure 1B, bottom). DAPI staining of synchronized meiotic samples showed that *dna2-md* timely underwent nuclear division (time course) and the frequency of nuclear division also reached the WT level (92% versus 95%) (Figure 1A). Moreover, *dna2-md* sporulated as efficiently as WT (93% in both WT and *dna2-md*) (Figure 1E). However, when the spore viability was analyzed through tetrad dissection, it dramatically decreased to 4.6% in *dna2-md* (95.9% in WT), indicating that Dna2 is required for normal meiosis (Figure 1F).

Inviable spores are mainly caused by aberrant chromosome segregation (56). We wondered whether *dna2-md* showed an increased frequency of chromosome mis-segregation. To this end, we integrated the red fluorescent protein (RFP) and the yellow fluorescent protein (YFP) driven by a spore-specific promoter into a pair of chromosomes 9, respectively (Supplementary Figure S2A; Materials and Methods). After meiosis, two spores show yellow fluorescence and the other two spores show red fluorescence in a tetrad. However, aberrant yellow/red fluorescence patterns represent mis-segregated chromosome 9. The frequency of chromosome 9 mis-segregation was defined as the number of tetrads with mis-segregated chromosome 9 divided by the number of total tetrads examined. In WT, the frequencies of chromosome segregation errors were very low in both meiosis I (MI) and meiosis II (MII) (a total of 1.7%). However, in *dna2-md*, the frequencies significantly increased to 3.2% and 24.6% in MI and MII, respectively, with the total mis-segregation frequency reaching 27.8% (Figure 1G and Supplementary Figure S2B). The high frequency of chromosome mis-segregation contributes to the extremely low spore viability in *dna2-md*.

### Both the nuclease and helicase activities of dna2 are required during meiosis I

The spore-specific assay showed that chromosome mis-segregation mostly occurred at MII but not MI in *dna2-md*. This may suggest that Dna2 is required for MII. However, it is also possible that the accumulated errors in MI lead to the high chromosome mis-segregation frequency in MII. We tried to determine the time window for Dna2 required for meiosis through two different systems.

First, Dna2 was degraded at different time points during meiosis. An auxin-inducible degron (AID) was fused to the C-terminus of Dna2 by the PCR-mediated method, hereafter *Dna2-AID* (31,32) (Figure 1H). When induced by copper, *Dna2-AID* expressed OsTIR1, an E3 ligase. Dna2-AID was ubiquitinated by an OsTIR1-containing E3 ubiquitin ligase complex and degraded by proteasomes in 15–30 min in the presence of auxin (IAA) and copper (32) (Supplementary Figure S3A). In synchronized cultures, Dna2-AID was efficiently removed by adding 2 mM IAA and 25 μM CuSO_4_ in SPM (Figure 1I). A low level of spore viability was observed when IAA and CuSO_4_ were added at 0 to 4 h in SPM (Figure 1J). However, the spore viability was significantly increased to 58.6% and 68.5% when IAA and CuSO_4_ were added at 5 and 6 h post SPM incubation, respectively (Figure 1J). Most cells have already reached or passed pachytene during this period (39,55,57). These results suggest that Dna2 is required at/before pachytene (5–6 h in SPM) to ensure successful meiosis and spore viability.

Second, one DNA2 native promoter was replaced by the *CLB2* promoter (to express Dna2 during vegetative growth but not meiosis) and the other DNA2 promoter was replaced by the *GAL1* promoter, hereafter *pCLB2-DNA2/pGAL1-DNA2* (Figure 1K). This strain also contained an estradiol-induced transcriptional cascade (58). In this system, Dna2 expression was induced timely and efficiently with 1 μM β-estradiol in 30 min (Figure 1L and Supplementary Figure S3B). When Dna2 expression was induced at 0 and 4 h in SPM, cells went through meiosis and the spore viability was 77.3% and 82.6%, respectively (Figure 1M). The spore viability is lower than that in WT strain maybe due to Dna2 induction slightly disturbs meiosis. While induction of Dna2 expression at 5h in SPM, the spore viability only showed a slight decrease compared to that of 0 or 4 h induction (65.9% vs 77.3% or 82.6%) (Figure 1M). However, late induction of Dna2 expression (at 6h or 7h in SPM) resulted in a dramatic decrease in spore viability (39.3% and 15.4%, respectively) (Figure 1M). These results further confirm that Dna2 is required during prophase I (most likely ∼5 h, i.e. around pachytene, and not late than 6 h in SPM) to ensure successful meiosis.

Dna2 has both nuclease and helicase activities (59–61). To investigate the requirements for Dna2 enzyme activities for proper meiosis, *DNA2*, *dna2-nd* (nuclease dead allele, *dna2E675A*) (60,62), and *dna2-hd* (helicase dead allele, *dna2R1253Q*) (62,63) under the control of the inducible *GAL1* promoter were separately introduced into *dna2-md*, which also carried an estradiol-induced transcriptional cascade. Thus, the transcription activity of the *GAL1* promoter can be induced by β-estradiol (Figure 1N). In the absence of β-estradiol, the *pGAL1-DNA2* strain produced 39% viable spores, while the *pGAL1-dna2-nd* and *pGAL1-dna2-hd* strains produced only 13% viable spores (Figure 1O, P). When 1 μM β-estradiol was added upon meiosis induction (0h in SPM), Dna2 in *pGAL1-DNA2*, dna2-nd in *pGAL1-dna2-nd*, and dna2-hd in *pGAL1-dna2-hd* were expressed at comparable levels (Figure 1O and Supplementary Figure S3C). The *pGAL1-DNA2* showed nearly the WT level of spore viability (87% versus 96%). The spore viability of *pGAL1-dna2-hd* was 51% (Figure 1P). These results indicate that both the nuclease and helicase activities of Dna2 are required for successful meiosis. We noted that *dna2-nd* almost completely prevented sporulation. However, the sporulation efficiency and spore viability reached 60% and 51% in *dna2-hd*, respectively. This may suggest the nuclease activity of Dna2 is more important than its helicase activity.

### Abundant accumulation of RPA in *dna2-md* through spo11-dependent and -independent manners

Given that the nuclease activity of Dna2 is stimulated by RPA in *in vitro* analysis (22,64), subsequently, we examined RPA localization in WT and *dna2-md* by chromosome spread of meiotic nuclei in combination with immunostaining using an anti-Rfa1 antibody (Figure 2A). The RPA signal was detected as individual foci in WT from 2 h in SPM, peaked at 4h (∼37 foci per nucleus), and then gradually decreased to a low level at 7 h (∼12 foci) when the majority of nuclei completed MI (Figure 2A, B). The dynamics of RPA foci reflect the dynamics of the formation and repair of meiotic DSBs (65).

In *dna2-md*, two types of morphologically different RPA signals were detected: individual RPA foci as in WT and long RPA lines rarely seen in WT (Figure 2A). These RPA lines were often observed at late time and it seemed that they resulted from many RPA molecules assembled on long ssDNA strands in *dna2-md*. In *dna2-md*, RPA foci were also detected at 2 h in SPM and the number gradually increased to a comparable level at 4h as in WT (38 versus 37 foci). However, in contrast to WT, the number of RPA foci continuously increased to a higher level at 6 h (∼55 foci) and was maintained at this level in *dna2-md* (Figure 2B). The number of RPA lines was hard to be quantified since they had different lengths and were usually tangled together. Alternatively, the frequency of nuclei bearing RPA lines was examined. The RPA lines were observed in several nuclei as early as 2 h in SPM, and the frequency of nuclei having RPA lines gradually increased to ∼70% at 6 h in SPM (Figure 2C).

Subsequently, to examine whether the accumulated RPA foci and/or lines in *dna2-md* were associated with meiotic recombination, they were further examined in *dna2-md spo11Y135F* and *spo11Y135F* mutants, where the DNA cleavage activity of Spo11 was abolished and thus the formation of meiotic DSBs did not occur (3). As expected, the RPA signal was rarely observed in the *spo11Y135F* mutant (Figure 2A, B). However, RPA foci were observed from 2 h in SPM and their number was increased to a high level at 4 h in *dna2-md spo11Y135F*, although it was lower than in *dna2-md* (18 versus 55 foci; Figure 2A, B). This suggests ∼1/3 RPA foci in the *dna2-md* form in a Spo11-independent manner. Moreover, nuclei with RPA lines were observed in *dna2-md spo11Y135F* as in *dna2-md* with a similar frequency (Figure 2C). These results suggest that RPA accumulates in *dna2-md* in Spo11-dependent and -independent ways.

The existence of RPA lines was confirmed by further analysis under a structured illumination microscope (SIM). In WT, only RPA foci were observed on Rec8 labelled chromosome axes. However, there were lots of RPA lines and it seemed that they extruded from Rec8-axes outward in *dna2-md* (Figure 2D). Further, RPA signal intensity was quantified at a per-nucleus level (Figure 2E). As expected, the intensity of the RPA signal in *dna2-md* was much higher than in WT. Interestingly, the RPA intensity in *dna2-md spo11Y135F* was also significantly higher than that in WT (Figure 2E). This is probably due to each RPA line containing a large number of RPA molecules.

Previous studies suggest that Dna2 is required for DNA replication in mitosis although one recent study showed the role of Dna2 in DNA replication is very limited (25,50,66,67). To characterize whether the accumulated RPA result from impaired pre-meiotic DNA replication in *dna2-md*, the native promoter of CDC6 was replaced by SCC1 to express Cdc6 only in mitosis but not in meiosis, named *cdc6-md*. In this strain, DNA replicates normally during mitotic growth but the pre-meiotic DNA replication is almost eliminated (30). The kinetics and efficiency of DSB formation and meiotic recombination in *cdc6-md* are also comparable with that in WT (30,68,69). Subsequently, we used chromosome spread of meiotic nuclei in combination with immunostaining to characterize the RPA signal in *cdc6-md* and *dna2-md cdc6-md* (Figure 2F). The number of RPA foci reached 55 in *dna2-md cdc6-md* as in *dna2-md*, which was significantly higher than WT (37 RPA foci) and *cdc6-md* (26 RPA foci) (Figure 2G). Furthermore, the percentage of cells with RPA lines in *dna2-md cdc6-md* was comparable to that in *dna2-md*, which was dramatically higher than that in *cdc6-md* (Figure 2H). These findings suggest that the abundantly accumulated RPA in *dna2-md* is not due to impaired pre-meiotic DNA replication. Consistently, the dynamics of pre-meiotic DNA replication in WT and *dna2-md* strains were comparable as revealed by flow cytometry analysis (Supplementary Figure S4).

### Accumulated RPA expands from DSBs to broader regions in *dna2-md*

The result of immunostaining showed that abundant RPA accumulated in *dna2-md*. To explore the effect of Dna2 on RPA distribution, we performed a genome-wide ChIP-seq in WT, *dna2-md* and *dna2-md spo11Y135F*. In WT, along with meiotic recombination progression, the number of RPA foci reaches the peak at 4h and dramatically decreases to a low level at 7h in SPM (Figure 2A, B; (65). Consistently, RPA was highly enriched around Spo11 oligo-enriched regions at 4h, and its enrichment was moderately decreased at 7h in WT when most DSBs were repaired. In *dna2-md*, a similar enrichment of RPA at Spo11-oligo sites was observed at 4h as in WT (Figure 3A, B, and Supplementary Figure S5). Intriguingly, in contrast to WT, the distribution of RPA was greatly expanded to broader regions from DSB hotspots at 7h in *dna2-md*, as shown at *ERG25* and *BAT1* DSB hotspots (Figure 3A, B, and Supplementary Figure S5). This result suggests that programmed DSB repair is defective in *dna2-md*. In *dna2-md spo11Y135F*, no obvious enrichment for RPA at Spo11 oligo sites was observed (Figure 3A, B, and Supplementary Figure S5), which is consistent with the absence of DSBs in this strain and further confirms that RPA enrichment at Spo11-oligo sites in WT and *dna2-md* depends on DSBs.

RPA enrichment at DSB flanks was further analyzed in detail at another four DSB hotspots in *dna2-md* (Figure 3C). Consistent with the preceding analysis, (i) RPA was highly enriched around all four DSB hotspots at 4 h in WT and *dna2-md* but not in *dna2-md spo11Y135F*, (ii) RPA enrichment around DSB hotspots was greatly reduced in WT but only slightly decreased in *dna2-md* at 7 h and (iii) RPA enrichment extended to broader regions flanking DSBs at 7 h in *dna2-md* but not in WT.

Subsequently, the correlation between RPA reads and Spo11 oligo counts was analyzed by dividing the genome of *S. cerevisiae* into 1 kb bins (Figure 3D–I). As expected, RPA reads and Spo11 oligo counts exhibited stronger correlations in WT than in *dna2-md* (*r* = 0.62 and 0.47 at 4h and 7h, respectively, in WT; *r* = 0.56 and 0.33 at 4 and 7 h, respectively, in *dna2-md*) since RPA expanded to broader regions from DSB sites in *dna2-md* (Figure 3D–G). However, no obvious correlation was detected between the RPA reads and Spo11 oligo counts in *dna2-md spo11Y135F* (*r* = 0.16 and 0.10 at 4 and 7 h, respectively) (Figure 3H, I). This is consistent with the Spo11-independent accumulation of RPA in *dna2-md spo11Y135F*.

### The amount of DSB formation, the length of DSB end resection, and the levels of crossover/non-crossover are comparable to WT, but DSB repair is defective in *dna2-md*

**Figure 4.**
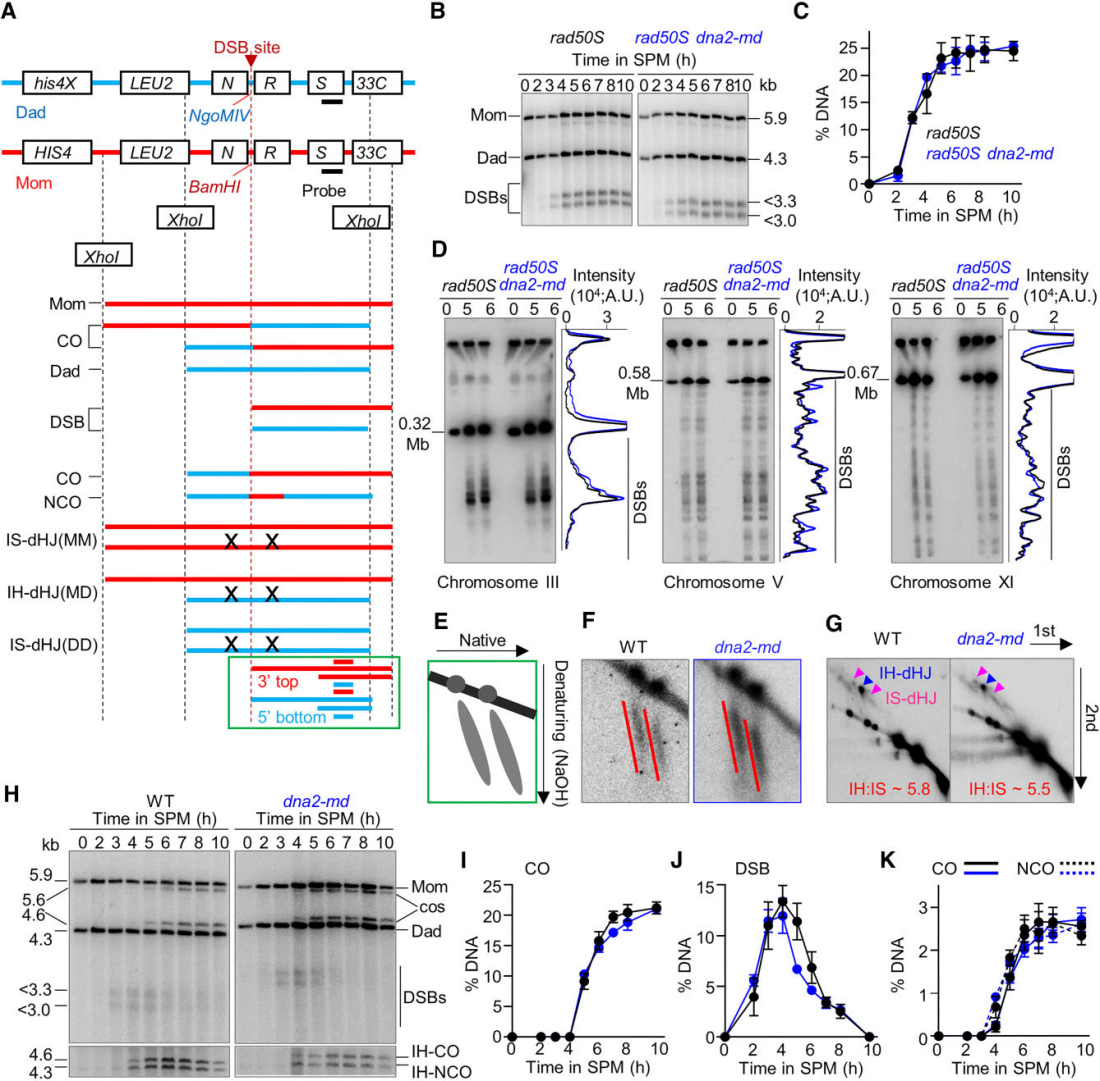
Seemingly normal DSB formation, end resection, and crossover/non-crossover formation at the *HIS4LEU2* hotspot in *dna2-md*. (**A**) The physical map of the *HIS4LEU2* hotspot and the probe position for recombination analysis. (**B**) Representative images to show DSBs at the *HIS4LEU2* in a *rad50S* background. (**C**) Quantification of (B) to show DSB levels. (**D**) Representative PFGE images and quantification (6 h) of DSBs intensity probed for chromosome III, V and XI in a *rad50S* background. (E, F) Cartoon (**E**) and representative images (**F**) to show DSB end resection detected on native/denaturing 2D gels in WT and *dna2-md*. (**G**) Representative 2D images to show IH-dHJ and IS-dHJ in WT and *dna2-md*. The ratios of IH-dHJ to IS-dHJ were indicated. (**H**) Representative images to show the detection of crossovers and DSBs (top panel), both crossovers and non-crossovers (bottom panel). (**I–K**) Quantification of (H) to show the levels of crossovers (I), DSBs (J) and crossovers and non-crossovers (K). Error bar, SEM (C, I–K, *n* = 3).

Abundant RPA accumulation detected cytologically and by ChIP-seq implies that the process of meiotic recombination and DSB repair is defective in *dna2-md*. Since meiotic recombination is initiated by programmed DSBs, we first examined the DSB formation at the well-characterized *HIS4-LEU2* hotspot by a standard southern hybridization analysis in WT and *dna2-md* (Figure 4A) (39). To avoid the DSB turnover issue and accurately measure the DSB level, the experiment was performed in a *rad50S* (K81I) background, which blocks the DSB end resection and accumulates DSBs. At *HIS4LEU2*, the accumulated DSB level reached ∼24% of total DNA in both WT and *dna2-md* (Figure 4B, C). DSBs were also examined with PFGE (Plus field gel electrophoresis) followed by southern hybridization at the chromosomal scale. Again, similar DSB levels and patterns were detected between *rad50S* and *dna2-md rad50S* on chromosomes III, V and XI, respectively (Figure 4D). These results suggest that Dna2 is not required for efficient DSB formation.

Soon after DSB formation, their 5′ ends are resected to generate 3′ ssDNA ends. The length of these 3′ ssDNA ends and thus resection level can be observed and evaluated on a native-denatured 2D gel (Figure 4A, green box, and Figure 4E) (42). For this purpose, the genomic DNA of WT and *dna2-md* was extracted and digested with Xho I. The digested DNA was analyzed by native electrophoresis in the first dimension and followed by denatured electrophoresis through sodium hydroxide in the second dimension (40,42). The resection length was analyzed after the southern blot. No obvious difference in DSB end resection was observed between WT and *dna2-md* (Figure 4F). This result suggests that Dna2 has no or little role in the 5′ end resection of meiotic DSBs. This is consistent with the previous finding that Exo1 is the main nuclease for DSB end resection, but Sgs1 has no contribution to this process in *S. cerevisiae* meiosis, although the Sgs1/Dna2 complex has an important role in mitotic DSB end resection (42,51,70,71).

Resected DSB ends invade homologous templates and are finally repaired as crossovers or non-crossovers. The recombination intermediate dHJ (double Holliday junction) and final products, crossovers and non-crossovers, can also be examined at the *HIS4LEU2* hotspot (Figure 4A) (39,40). Both the inter-homolog and inter-sister double Holliday junctions (IH and IS-dHJ) were detected on 2D gels in combination with southern hybridization. Quantification analysis showed the ratio of IH-dHJ to IS-dHJ was ∼6:1 in both WT and *dna2-md* (Figure 4G). The 1D gels in combination with southern hybridization showed ∼20% crossover DNA and ∼1:1 ratio of crossover to non-crossover in both WT and *dna2-md* (Figure 4H–K). These results suggest that Dna2 is not required for the formation of crossovers or non-crossovers, at least at the *HIS4LEU2* hotspot.

Crossovers were also examined at another hotspot, *ERG1*, and no obvious difference was observed between WT and *dna2-md* (Supplementary Figure S6A, B) (72). Therefore, it is likely that crossovers form normally in *dna2-md*. Consistent with this idea, both WT and *dna2-md* showed similar numbers of crossover-associated Zip3 foci at pachytene (60 versus 61.7 per nucleus) (Supplementary Figure S6C, D), which label patterned crossovers in budding yeast meiosis (37,73,74).

Synapsis is another hallmark of meiosis. The possible role of Dna2 in synapsis was also examined. To avoid the possible asynchronization issue, an *ndt80Δ* was introduced into the *dna2-md* strain to arrest cells at pachytene (75). The synapsis was evaluated by the morphology of the SC central element Zip1 (35,65). During meiosis, the morphology of Zip1 can be divided into four classes: no signal (Class I), dotty (Class II), partially elongated (Class III), and fully elongated (Class IV). In *ndt80Δ*, when Zip1 was visualized in spread nuclei collected at 8 h in SPM, 98% of the nuclei had a detectable Zip1 signal. The Class I–Class IV nuclei took up 2%, 9%, 19% and 70%, respectively (Supplementary Figure S6E, F). Similar frequencies of nuclei for each class were observed in *dna2-md ndt80Δ* like in WT (4%, 6%, 23% and 66%, respectively) (Supplementary Figure S6E, F). Therefore, these results indicate that Dna2 has no or little role in synapsis.

**Figure 5.**
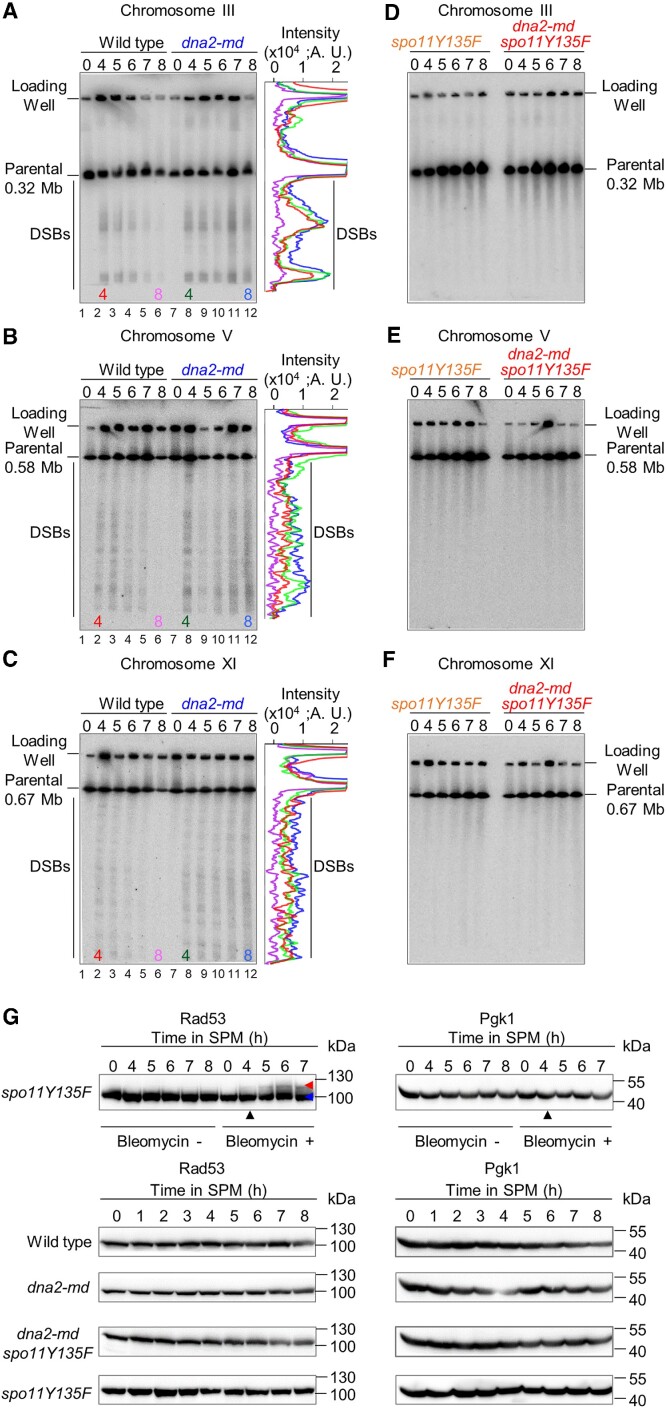
Defective repair of meiotic DSBs in *dna2-md*. (A–C) Representative PFGE Southern blot (left panel) and quantification (at 4 and 8 h) of DSB intensity (right panel) probed for chromosome III (**A**), chromosome V (**B**) and chromosome XI (**C**) in WT and *dna2-md*. (D–F) Representative PFGE Southern blot probed for chromosome III (**D**), chromosome V (**E**) and chromosome XI (**F**) in *spo11Y135F* and *dna2-md spo11Y135F*. (**G**) Rad53 phosphorylation examined by western blot. Rad53 phosphorylation was observed in samples treated with bleomycin. Bleomycin was added at SPM 4 h (indicated by the black triangle). The phosphorylated and un-phosphorylated Rad53 were indicated by the red and blue triangles, respectively. Pgk1 was used as a loading control.

Taken together, our findings suggest that accumulated RPA have a limited effect on the amount of DSB formation, the length of DSB end resection, the levels of crossover/non-crossover, and synapsis in *dna2-md*. However, the high frequency of chromosome mis-segregation and extremely low spore viability imply that accumulated RPA likely impair the meiotic DSBs repair in *dna2-md*. To confirm this idea, PFGE in combination with southern hybridization was performed to examine the extent of DSB repair on three representative chromosomes with different lengths, III, V, and XI. At 0 h in SPM, almost no DNA fragment was detected (Figure 5A–C, lane 1, ‘0 h’). Abundant DNA fragments were observed at 4 h in SPM due to meiotic DSBs in WT (Figure 5A–C, lane 2). The abundance of DNA fragments was gradually decreased, accompanied by DSB repair, and they were barely detectable at 8 h in SPM (Figure 5A–C, lanes 3–6). A similar level and pattern of DNA fragments were observed at 4h in *dna2-md* as in WT (Figure 5A–C, lane 8, red versus green curves), suggesting DSB formation is not affected in *dna2-md*. However, there was still a high level of DNA fragments at 8h in *dna2-md* (Figure 5A-C, lane12, purple versus blue curves), which indicates defective DSB repair. This observation is consistent with the Spo11-dependent accumulation of RPA in *dna2-md*. To further confirm the defective DSB repair in *dna2-md*, we performed the comet assay (Supplementary Figure S7A–C). At 4h in SPM, most nuclei in both WT and *dna2-md* (86.6% versus 84.0%) showed obvious comet tails, indicating severe DNA damage. At 7 h in SPM, comet tails were only observed in a few WT nuclei (6.9%), implying efficient DSB repair. However, a high frequency of nuclei with obvious comet tails was still observed at 7 h as at 4 h in *dna2-md* (86.7% versus 84.0%). This is consistent with the accumulation of RPA and thus impaired DSB repair in *dna2-md*.

As expected, no/little DNA fragments were observed in either *dna2-md spo11Y135F* or *spo11Y135F* mutant, where the occurrence of programmed DSBs is abolished (Figure 5D–F). This data further supports the idea that RPA accumulation in *dna2-md spo11Y135F* is independent of Spo11-mediated DSBs. In addition, when the nuclear division (time course), spore viability, and the frequency of chromosome mis-segregation were analyzed in *dna2-md spo11Y135F* and *spo11Y135F*, no significant differences were found between these two mutant strains, implying that no Spo11-independent DSBs occur in *dna2-md spo11Y135F* (Supplementary Figure S8A–C). Exogenous DNA lesions trigger Rad53 phosphorylation, which can be seen as altered electrophoretic mobility in *spo11Δ* meiotic cells (Figure 5G, top panel) (76). Consistent with our above idea that there are no Spo11-independent DSBs in *dna2-md* and *dna2-md spo11Y135F*, no Rad53 phosphorylation signal was detected in these strains (Figure 5G, bottom panel). Our results collectively reveal that defective meiotic DSB repair occurs in *dna2-md*.

### Dna2 removes RPA filaments generated during meiotic recombination-associated DNA synthesis to maintain genome integrity

**Figure 6.**
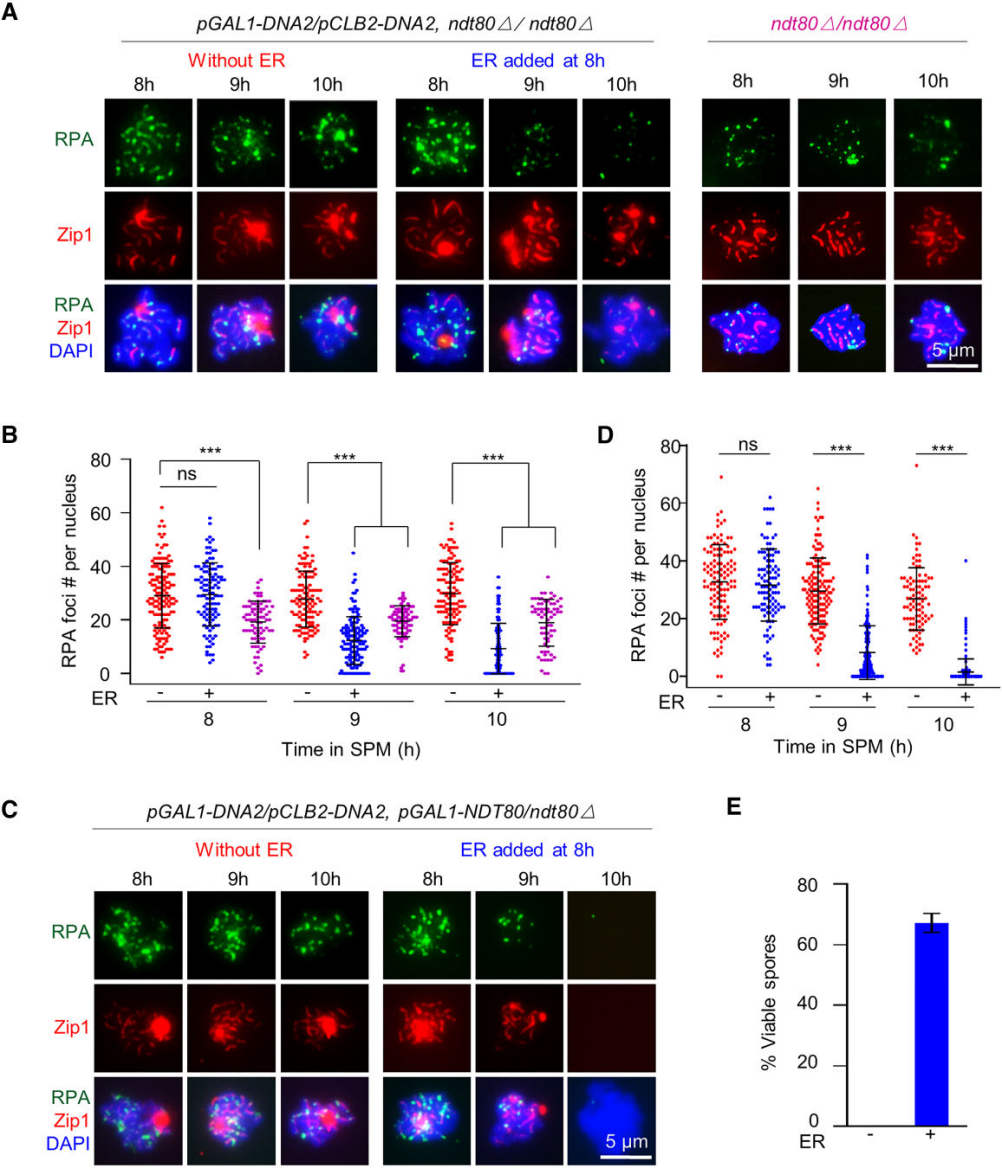
The pachytene induction of Dna2 efficiently removes accumulated RPA. (A, B) Representative images (**A**) and quantification (**B**) to show induction of Dna2 expression removed accumulated RPA in *ndt80Δ*. Sample size, *n* = 151, 112, 79, 123, 140, 86, 101, 111 and 69 nuclei, respectively. (C–E) Representative images (**C**) and quantification (**D**) to show that simultaneous induction of Dna2 and Ndt80 expression removed accumulated RPA and largely rescued spore viability (**E**). Sample size, *n* = 118, 109, 148, 175, 94 and 203 nuclei, respectively (D). 96 tetrads were examined for each strain (E). Error bar, SD (B, D) or 95% confidence interval (E). Scale bar, 5 μm (A, C). Two-tailed Student's *t*-test (B, D); ns (not significant), ** (*P* < 0.01), and *** (*P* < 0.001).

Our above results suggest apparently normal crossover and non-crossover formation in *dna2-md*. However, the PFGE result showed most DSBs were unrepaired. This paradox can be reconciled if new DNA is synthesized from DSB ends and extended longer than the restriction cleavage sites used for 1D and 2D gel analysis. This would give seemingly intact crossovers and non-crossovers at a local scale, however, simultaneously produce long DNA flaps which would be visualized as long RPA lines and unrepaired ssDNA breaks at a large scale if they are not timely removed (see Discussion) (17,22). If so, induced expression of Dna2 at pachytene may remove these accumulated RPA in *dna2-md*. To test this idea, *ndt80△* was introduced into the *pCLB2-DNA2/pGAL1-DNA2* strain to arrest cells at pachytene (Figure 6A). As expected, a considerable number of RPA foci was observed in *ndt80Δ*, and abundant RPA foci and lines were observed and maintained in pachytene-arrested nuclei in *pCLB2-DNA2/pGAL1-DNA2 ndt80Δ* (Figure 6A, B). When β-estradiol was added to induce Dna2 expression in the latter strain, almost all RPA signal disappeared quickly (Figure 6A, B). Further, to examine whether the removal of accumulated RPA at pachytene could rescue spore viability, we simultaneously induced the expression of Dna2 and Ndt80 after pachytene arrest, and thus the strain could complete meiosis (Figure 6C, D). In this case, RPA was dramatically decreased and the spore viability reached up to 67% (Figure 6E). These results suggest that Dna2 induction at pachytene can largely remove accumulated RPA and rescue spore viability. The lower spore viability in this strain compared with WT (67% versus 96%) may be due to the late expression of Dna2, which cannot timely remove RPA and thus prevent the timely repair of DSBs before pachytene exits. These results support the proposal that Dna2 removes ssDNA-RPA filaments at/before pachytene for DSB repair and thus spore viability.

**Figure 7.**
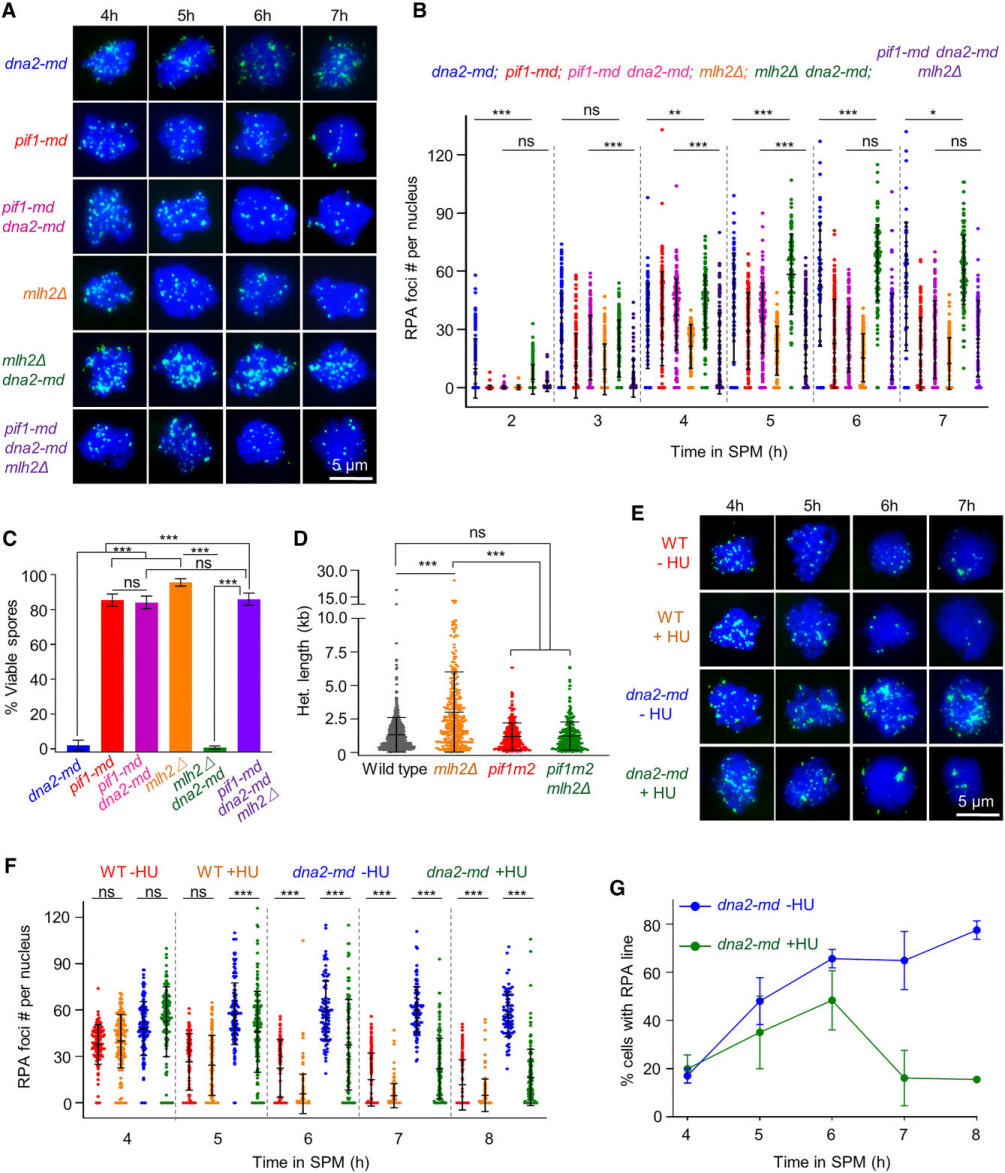
RPA accumulation requires new DNA synthesis during meiotic recombination. (A, B) Representative images (**A**) and quantification (**B**) to show RPA in *dna2-md, pif1-md*, *pif1-md dna2-md*, *mlh2Δ, mlh2Δ dna2-md* and *pif1-md dna2-md mlh2Δ*. Sample size, *n* = 143, 62, 58, 50, 113, 94, 165, 175, 179, 160, 168, 91, 119, 169, 192, 148, 140, 113, 85, 158, 238, 153, 120, 100, 55, 176, 208, 161, 106, 112, 54, 140, 151, 153, 106 and 107 nuclei, respectively. The *dna2-md* data were duplicated from Figure 2B. (**C**) Pif1 depletion rescued the spore viability of *dna2-md*. For each strain, 96 tetrads were examined. (**D**) The heteroduplex DNA (hDNA) tract lengths of recombination products (in *msh2Δ* background). The re-sequenced genomic data are obtained from NCBI under SRA accession number SRP075437 (16). *pif1m2*: a point mutation at the second ATG of the *PIF1* gene causes the loss of Pif1 function in the nucleus while keeping its function in mitochondria (16). (**E**) Representative images to show the effect of HU on RPA accumulation in WT and *dna2-md*. (F, G) Quantification of (E) to show the number of RPA foci per nucleus (**F**) and the percentage of cells with RPA lines (**G**). Sample size, *n* = 132, 136, 148, 137, 128, 144, 123, 154, 132, 141, 93, 106, 151, 128, 109, 136, 120, 128, 80 and 131 nuclei, respectively. Error bar, SD (B, D, F), SEM (G) or 95% confidence interval (C). Scale bar, 5 μm (A, E). Two-tailed Student's *t*-test (B, D, F) or Two proportion Z-test (C); ns (not significant), * (*P* < 0.05), ** (*P* < 0.01) and *** (*P* < 0.001).

Subsequently, we further explored whether the accumulated RPA in *dna2-md* result from meiotic recombination-associated DNA synthesis. Pif1 is known to stimulate Pol δ strand displacement activity during homologous recombination in mitosis, and a recent study discovered that Pif1 is also involved in producing long DNA synthesis tracts during meiotic recombination in *mlh2Δ* (16,17,77,78). The meiosis-specific depletion of Pif1 (*pif1-md*) would decrease the activity of Pol δ, resulting in less-extended D-loops and fewer ssDNA-RPA filaments, while, the removal of Pif1 inhibitor Mlh2 would over-stimulate Pol δ, resulting in over-extended D-loops and more ssDNA-RPA filaments in *dna2-md*. These predictions were confirmed by the fact that fewer RPA accumulation in *pif1-md dna2-md* but more RPA accumulation in *dna2-md mlh2Δ* were observed (Figure 7A, B). Moreover, spore viability was 84% in *pif1-md dna2-md* and 86% in *pif1-md dna2-md mlh2Δ* (85% in *pif1-md*) but only 0.8% in *dna2-md mlh2Δ* (95% in *mlh2Δ*) (Figure 7C). Consistent with the above proposal, the mean length of heteroduplex DNA (hDNA) tracts is decreased although slightly in a *pif1* mutant *pif1m2* (1.2 kb) and *pif1m2 mlh2Δ* (1.2 kb), while it is dramatically increased in *mlh2Δ* (3.0 kb) compared with that in WT (1.3 kb) when they were analyzed with publicly available re-sequenced spores (Figure 7D).

Further, we analyzed whether the accumulation of RPA requires new DNA synthesis by adding a ribonucleotide reductase inhibitor, hydroxyurea (HU), at 4 h in SPM to deplete dNTPs and thus block DNA synthesis for DSB repair. The accumulation of RPA reached its peak at ∼4 h in SPM and then gradually decreased along with DSB repair in WT, however, the RPA signal reached a much higher level in *dna2-md* at late time (Figures 2A, B and 7E–G). In WT cells, the addition of HU at 4 h resulted in a faster decrease of the RPA signal. In *dna2-md* cells, a quick decrease in RPA signal (the number of RPA foci and the frequency of cells with RPA lines) was observed in the presence of HU although it was further accumulated in the absence of HU (Figure 7E–G). The different RPA dynamics caused by HU probably result from the combined effects of (i) the depletion of dNTPs and block of DNA synthesis, which prevents further accumulation of RPA, and (ii) RPA displacement by Rad51/Dmc1. Taken together, our findings support the idea that Dna2 is required for removal of ssDNA-RPA filaments formed during meiotic recombination-associated DNA synthesis.

### Spo11-independent RPA distribution is regulated by dna2

Our cytological observations revealed that a considerable level of RPA signal was Spo11-independent in *dna2-md*. Detailed analysis of our ChIP-seq results showed that ∼20% of RPA peaks were distributed outside of Spo11-oligo regions and at centromeres and tRNA regions in WT (Supplementary Figure S9A). Interestingly, a large fraction of RPA was enriched in type I and II (but not III-V) retrotransposons in *dna2-md* (Supplementary Figure S9A, B). Moreover, the enrichment of RPA at retrotransposons was detected more clearly at 7h than at 4h (Supplementary Figure S9A, B). However, almost no RPA enrichment was observed at retrotransposons in WT at either 4 or 7 h (Supplementary Figure S9A, B). These results suggest that Dna2 prevents RPA accumulation at types I and II retrotransposons. This role of Dna2 is independent of Spo11-mediated DSBs since similar RPA enrichment was observed in the *dna2-md spo11Y135F* double mutant. Compared with WT, RPA also tended to enrich at telomeres in *dna2-md* and *dna2-md spo11Y135F* (Supplementary Figure S9C, left panel). These findings imply that, as in mitosis, Dna2 plays a critical role at telomeres in meiosis (79,80). We also noted that RPA was enriched at rDNA in *dna2-md* and *dna2-md spo11Y135F* but not in WT (Supplementary Figure S9C, right panel). We observed a number of RPA peaks in *dna2-md spo11Y135F* enriched at regions that are Spo11 oligo hotspots in WT, especially at 4h in SPM. These results suggest that Dna2 also regulates Spo11-independent RPA distribution.

## DISCUSSION

### Dna2 removes ssDNA-RPA filaments formed during meiotic recombination-associated DNA synthesis

**Figure 8.**
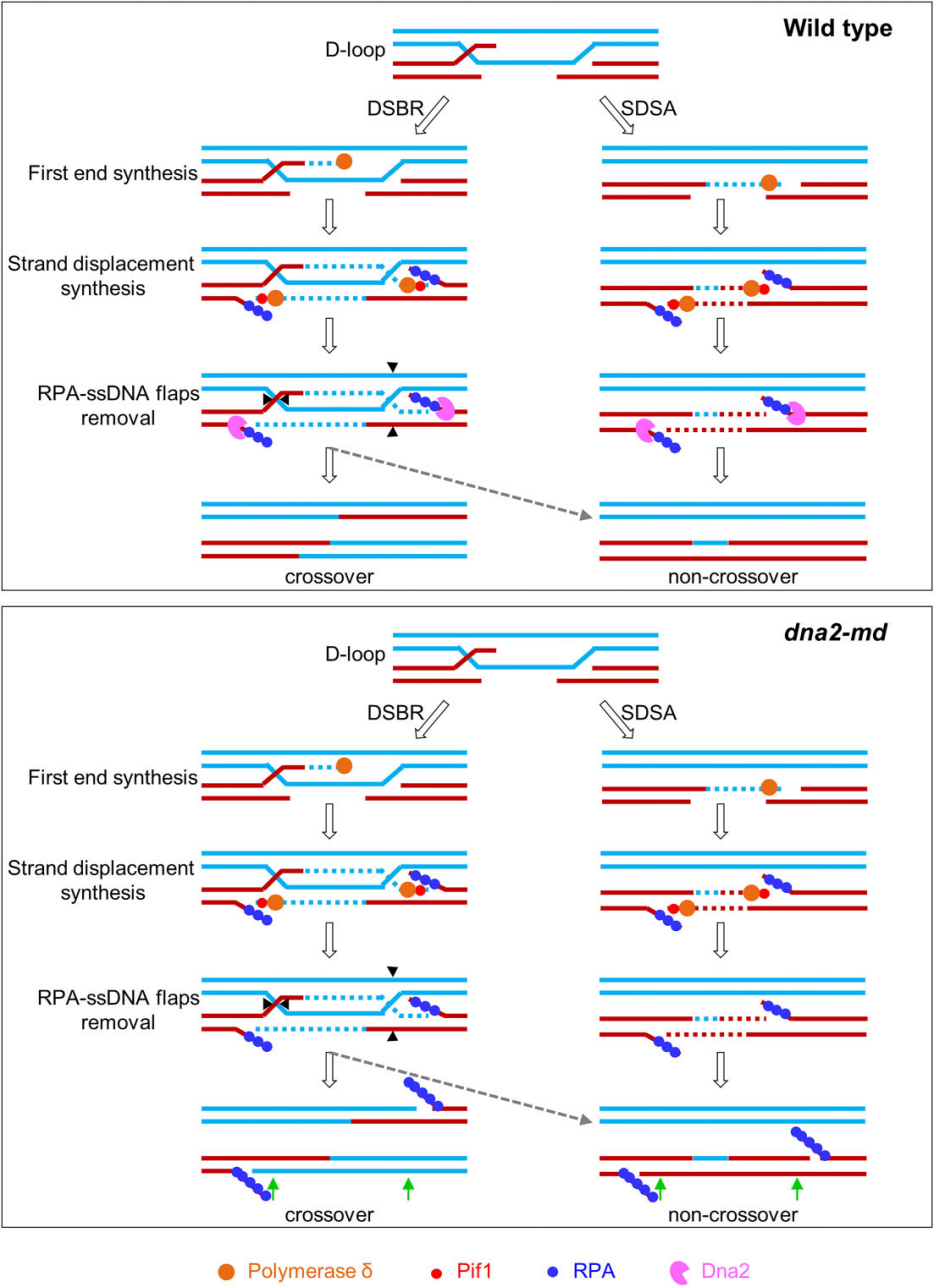
The possible role of Dna2 in removing ssDNA-RPA filaments in the meiotic DSB repair model. Please see Supplementary Figure S1 and the text for a detailed description of the DSB repair model. In this model, Pol δ probably catalyzes new DNA synthesis and thus the formation of ssDNA through its strand displacement activity. Pif1 functions as an activator of Pol δ and is also involved in this process. In WT, ssDNA-RPA filaments are removed by Dna2 and then the nick is ligated for complete meiotic DSB repair. However, in *dna2-md*, the persistent ssDNA-RPA filaments block nick ligation and (1) result in extended DNA synthesis and thus ‘seemingly normal’ crossovers and non-crossovers when examined at a short scale (e.g. on 1D and 2D gels at hotspots), and (2) produce DNA fragments seen on PFGE due to closely spaced DNA nicks on opposing strands, newly synthesized DNA strand encounters a nick on the template strand, or two synthesized DNA strands collide. The green arrows at the bottom indicate the restriction enzyme cleavage sites used to examine DSBs and recombination at a hotspot.

We showed that Dna2 is essential for meiosis. When Dna2 is meiosis-specifically depleted (*dna2-md*), RPA dramatically extends from DSB sites to broad regions, resulting in unpaired DSBs as revealed by PFGE and abundant accumulation of RPA, ultimately chromosomes show a high frequency of mis-segregation and spores are inviable. Consistent with a putative role of Dna2 in cleaving ssDNA flaps during mitosis, the Dna2 nuclease-dead mutant showed the same defects as the *dna2-md*, and pachytene induction of Dna2 can effectively remove accumulated RPA and restore spore viability. Therefore, we propose that Dna2 prevents/removes ssDNA-RPA filaments formed during meiotic recombination-associated DNA synthesis (Figure 8). The role of Dna2 in this model is also consistent with its role in mitosis to cleave ssDNA flaps during lagging strand synthesis (50). Our model is further supported by the following evidence. (1) The accumulation of RPA requires new DNA synthesis since the addition of HU to deplete dNTPs prevents the abundant accumulation of RPA signal. (2) Depletion of the Pol δ activator Pif1 in *dna2-md* prevents RPA accumulation and restores spore viability. Moreover, removing a Pif1 inhibitor, Mlh2, which is predicted to cause D-loop over-extension, results in RPA over-accumulation in *dna2-md*. Additionally, the mean hDNA tract length in *pif1m2* (1.2 kb) and *pif1m2 mlh2Δ* (1.2 kb) is comparable to WT (1.3 kb), while it is dramatically increased in *mlh2Δ* (3.0 kb). However, we also found that *dna2-md* shows the normal level of DSBs and meiotic recombination progresses normally to generate WT levels of recombination products, i.e. COs and NCOs, when examined by Southern blot at *HIS4LEU2* and *ERG1* hotspots. This result seems to be contradicted with our above proposal. However, this paradox can be easily reconciled as discussed below.

During meiotic recombination, Pol δ catalyzes DNA synthesis (16). When this newly synthesized strand encounters the second DSB end, the strand displacement activity in combination the polymerase activity of Pol δ generates flapped ssDNA which is coated by RPA to form ssDNA-RPA filaments. Dna2 removes/prevents ssDNA-RPA filaments for timely nick ligation to restore genome integrity. However, in the absence of Dna2, continuously DNA synthesis causes the newly synthesized strands continuously grow and displace the parental strands, which ultimately results in the generation of longer ssDNA-RPA filaments and the migration of DNA nicks far away from the original DSB sites (Figure 8). In Southern blot, a small region spanning DSBs (∼3 kb on each side of *HIS4LEU2* and ∼5 kb at *ERG1*) is analyzed to detect DSBs and recombination products (Figure 4A) (72). When newly synthesized DNA strands extend out of the analyzed regions, seemingly repaired DSBs (and recombination products) are detected on 1D and 2D gels (Figure 4H–K, and Supplementary Figure S6A, B). Consistently, CO-associated Zip3 foci, assumed to mark dHJ, are also observed at the WT level (Supplementary Figure S6C, D). However, these persistent ssDNA-RPA filaments prevent nick ligation and thus chromosome fragments are observed by PFGE (Figures 5A–C).

Although Dna2 plays an important role in DNA replication during mitosis, our study does not show an observable defect in pre-meiotic DNA in *dna2-md*. (i) The dynamics of pre-meiotic DNA replication in WT and *dna2-md* are comparable. (ii) When pre-meiotic DNA replication is abolished in *dna2-md cdc6-md*, abundant RPA accumulates as in *dna2-md*. This suggests the recombination defects in *dna2-md* are not resulted from defects in pre-meiotic DNA replication. (iii) The abundant accumulation of RPA signal requires *de novo* DNA synthesis during meiotic recombination as revealed by the HU experiments. However, our results do not mean that Dna2 has no role in pre-meiotic DNA replication since a low level of Dna2 in *dna2-md* may be enough for this process.

In summary, our findings suggest that Dna2 mainly removes/prevents ssDNA-RPA filaments generated from newly synthesized DNA during meiotic recombination and accumulated filaments are a roadblock that leads to the DSB repair defect. However, there are still important issues that remain elusive and are worth further investigation.

1. Our findings in combination with previous reports suggest that Pif1 stimulates Pol δ to generate DNA flaps, which are coated by RPA, and then these RPA filaments have to be removed by Dna2 (16). In the absence of Pif1, abundant RPA accumulation is inhibited and the spore viability is rescued to nearly WT level in *dna2-md pif1-md*. Since *pif1* depletion can also rescue the lethality of *dna2Δ* in mitosis (22,62), one possible interpretation for our result is that inviable spores in *dna2-md* are due to the mitotic defects after meiosis. However, this possibility is less likely because (1) Dna2 is expressed in mitosis in *dna2-md* and (2) *dna2-md* does show severe meiotic defects (above discussion). Therefore, we prefer another interpretation that in the absence of Pif1, the activity of Pol δ is partially inhibited, and thus Dna2 is not necessary during meiosis. However, this raises the interesting question of why meiosis requires Pif1 to generate long DNA flaps and then requires Dna2 to remove them.
2. Our findings suggest that Pif1, Pol δ, and Dna2 are responsible for the formation and removal of ssDNA-RPA filaments during meiotic recombination-associated DNA synthesis. However, it is unclear whether ssDNA-RPA filaments form at the first or the second DSB end, or both. This is most likely related to whether Pol δ mediates first or second strand synthesis, or both, though evidence suggests that Pif1 may only be involved in the first-end synthesis (16). Another critical question is how Pol δ collaborates with Pif1, RPA, Dna2, and other factors to timely resolve ssDNA-RPA filaments and maintain genome integrity.
3. The nuclease-dead Dna2 mutant shows a very high level of RPA accumulation and produces inviable spores as *dna2-md* (Figure 1P and Supplementary Figure S10A, C, D). This is consistent with our proposal that Dna2 removes ssDNA-RPA filaments via its nuclease activity. Interestingly, the helicase-dead mutation of Dna2 also showed a moderate level of accumulation of RPA and a lower level of spore viability (Figure 1P and Supplementary Figure S10B–D). This raises the issue that why the helicase activity of Dna2 is also required for normal meiosis in *S. cerevisiae*. The physiological function of Dna2 helicase activity remains also elusive in mitosis. It seems that the Dna2 helicase activity is required at a small subset of troubled replication forks although it is not required for bulk DNA replication in mitosis (19,63,81). In Dna2 helicase-dead cells in mitosis, a low level of Rad53 phosphorylation was detected probably due to trouble DNA replication caused DNA damages and this phosphorylation is suppressed by deletion of the DNA damage checkpoint mediator Rad9 (63). Therefore, one possibility is that dna2-hd has a pre-meiotic DNA replication defect as that in mitosis. However, this is less likely since our results suggest Dna2 is not required for pre-meiotic DNA replication (see above discussion). Moreover, we failed to detect the phosphorylation of Rad53 in *dna2-md* and *dna2-md spo11Y135F* (Figure 5G). Another possibility is that the full activity of nuclease requires its helicase activity for an unknown reason. This is also less likely since the helicase-dead mutant used in this study shows full nuclease activity in *in vitro* studies (63). It is also possible that Dna2 may use its helicase activity to resolve a short piece of the double-stranded DNA at the base of the RPA filaments for more efficient cleavage. A similar process likely occurs during mitotic recombination, where Dna2 requires the helicase activity of Sgs1 for efficient DSB end resection (51,71).
4. During meiosis, defects in homologous recombination/synapsis trigger the meiotic prophase checkpoint, resulting in cell cycle arrest for repair and rescue or cell apoptosis (82–85). The meiotic checkpoint machinery shares many components with the canonical DNA damage response pathway, the most important components among which are the evolutionarily conserved sensor kinases, ATM/Tel1 and ATR/Mec1 (83,85–88). Activated ATM/Tel1 and ATR/Mec1 phosphorylate a large set of substrates to either directly implement the checkpoint response or transmit the signal to downstream effectors depending on the nature of the defect and the local environment (83,84). In *dna2-md*, no defect in synapsis is observed, but RPA-coated ssDNA is accumulated to a high level in meiosis. RPA-coated ssDNA is a classical signal triggering DNA damage response in both mitosis and meiosis (7,89–91). However, it does not activate the meiotic checkpoint and nuclei divide timely resulting in inviable spores in *dna2-md*. How is this possible? Different from other recombination/synapsis-defective mutants (e.g. *dmc1Δ*, *zip1Δ, hop2Δ, mus81Δ*) which show aberrant recombination at various steps and/or synapsis (92–95), *dna2-md* shows timely and normal synapsis and recombination at each step (at least at a local scale, also see above discussion): DSB formation, end resection, homolog bias, importantly normal levels of recombination products when examined at *HIS4-LEU2* by Southern blot. This means that the key events monitored by the meiotic recombination checkpoint progress normally in *dna2-md*. Consistently, the dynamics of Mek1 phosphorylation and dephosphorylation in *dna2-md* are comparable to that in WT, which is totally different from the persistent phosphorylation of Mek1 as seen in *dmc1Δ* (Supplementary Figure S11A, 76). In addition, the Mek1-dependent phosphorylation of Red1 is required to activate the pachytene checkpoint and its dephosphorylation is necessary for pachytene exit (96,97). Similar dynamics of Red1 phosphorylation were also observed between WT and *dna2-md*, however, a high level of phosphorylated Red1 was persistent in *dmc1Δ* (Supplementary Figure S11B). This suggests the accumulated RPA in *dna2-md* is different from that in *dmc1Δ* and probably also other recombination/synapsis-defective mutants. Therefore, the accumulated RPA filaments do not activate the checkpoint in *dna2-md*.

### Does dna2 function in other processes in meiotic cells?

Dna2 is required for DNA replication and DSB end resection in mitosis (51,98). However, one recent study shows that the function of Dna2 in DNA replication is extremely limited (25). Therefore, the role of Dna2 in mitotic DNA replication is still controversial. In this study, it seems that Dna2 is not required for pre-meiotic DNA replication as revealed by the results of flow cytometry and dynamics of nuclear division. However, we cannot rule out the possibility that the residual Dna2 performs these functions efficiently in *dna2-md*. Meanwhile, the results of native-denatured 2D gel in combination with Southern hybridization indicate that Dna2 is not required for meiotic DSB resection, which is consistent with previous reports that Exo1 is the core nuclease for long-term DSB resection in *S. cerevisiae* meiotic cells (42,70). Taken together, we reveal that unlike in mitosis, Dna2 has no or only very limited roles in DNA replication and DSB end resection during meiosis in *S. cerevisiae*.

In the absence of both meiotic DSBs and Dna2 (*dna2-md spo11Y135F*), a considerable level of RPA is also observed by immunostaining. Interestingly, the ChIP-seq analysis reveals that RPA accumulates at types I and II retrotransposons in *dna2-md* and *dna2-md spo11Y135F*, suggesting that Dna2 actively prevents RPA accumulation in these regions in a Spo11-independent manner. *S. cerevisiae* contains five families of LTR (long terminal repeat) retrotransposons, including Ty1, Ty2, Ty3, Ty4, and Ty5 elements, which contribute to approximately 3% of the genome (99–101). These retrotransposons share the basic structure: two direct terminal repeats flank the *TYA* and *TYB* encoding genes, which are analogous to the gag and pol genes of retroviruses (100,102). It is currently unknown why and how RPA only accumulates at types I and II but not at other types of retrotransposons regardless of the presence or absence of meiotic-DSBs. We can imagine several possibilities. (i) For the *S. cerevisiae* S288C genome, there are 32 copies of Ty1 and 13 copies of Ty2, but only 2 copies of Ty3, 3 copies of Ty4, and 1 copy of Ty5 (100). The high copy numbers of Ty1 and Ty2 may contribute to the preferential RPA accumulation. (ii) Although there are no meiotic DSBs and the integrity of genomic DNA is not compromised in *dna2-md spo11Y135F*, it is still possible that a very low level of un-programmed DSBs occurs at types I and II retrotransposons since they are unstable, especially in the absence of Dna2. (iii) Dna2 depletion improves the cDNA stability of retrotransposons in mitotic nuclei (62). In mitosis, several mutants associated with DNA replication and repair, including Fen1, Sgs1, Rad57, Rad2, Rad50, Mre11, and Tel1, have increased stability of retrotransposon cDNA but not RNA (103–105). It is very likely that increased cDNA levels also occur in meiotic nuclei to promote the formation of ssDNA-RPA flaps. (iv) It is also possible that in Dna2 depleted meiotic nuclei, there is increased transcription at these retrotransposons and the ssDNA is directly coated by RPA to generate ssDNA-RPA filaments, or that increased transcription causes DNA damage, which further recruits RPA binding. (v) Another possibility is that in Dna2-depleted meiotic nuclei, RNA transcripts bind to DNA templates (especially when transcriptional activity is high) and generate R-loops, leaving displaced ssDNA that is then coated by RPA (106). Further investigations are required to figure out how Dna2 regulates RPA enrichment at Ty1 and Ty2 retrotransposons.

In the depletion of Dna2, RPA also tends to be enriched at telomeres and rDNA. As a result, Dna2 is required in both meiosis and mitosis to prevent ssDNA accumulation and thus maintain genome stability (79,80). Additionally, a fraction of RPA in *dna2-md spo11Y135F* locates in regions that are Spo11-oligo hotspots in WT. These regions are most likely actively transcribed and easily form ssDNA. Therefore, Dna2 may also be involved in other processes to prevent Spo11-independent RPA accumulation.

## Supplementary Material

gkad537_Supplemental_File

## Data Availability

Data used in the paper are present in the paper and/or the Supplementary Materials. The RPA ChIP-seq data of WT, *dna2-md* and *dna2-md spo11Y135F* are deposited in the NCBI Gene Expression Omnibus (GEO) database under the accession number GSE217628. Data used for the analysis of hDNA tract lengths are obtained from NCBI under SRA accession number SRP075437 (16).
