# Supplementary material for: Dna2 removes toxic ssDNA-RPA filaments generated from meiotic recombination-associated DNA synthesis
Source: Nucleic Acids Res. 2023 Jun 23;51(15):7914–35. doi: 10.1093/nar/gkad537 (PMC10450173; doi:10.1093/nar/gkad537)
Supplement: gkad537_Supplemental_File [file gkad537_supplemental_file.pdf]

SUPPLEMENTARY INFORMATION

Dna2 removes toxic ssDNA-RPA filaments generated from meiotic recombination-associated DNA synthesis

Binyuan Zhai[1*,†], Shuxian Zhang[2,†], Bo Li[3], Jiaming Zhang[2], Xuan Yang[2], Yingjin Tan[2], Ying Wang[1], Taicong Tan[2], Xiao Yang[2,4,5,6], Beiyi Chen[2,7], Zhongyu Tian[2,7], Yanding Cao[2], Qilai Huang[3], Jinmin Gao[1], Shunxin Wang[2,4,5,6*], Liangran Zhang[1,7*]

[1] Center for Cell Structure and Function, Shandong Provincial Key Laboratory of Animal Resistance Biology, College of Life Sciences, Shandong Normal University, Jinan, Shandong 250014, China

[2] Center for Reproductive Medicine, Cheeloo College of Medicine, Shandong University, Jinan, Shandong 250012, China.

[3] Shandong Provincial Key Laboratory of Animal Cell and Developmental Biology, School of Life Sciences, Shandong University, Qingdao, Shandong 266237, China

[4] National Research Center for Assisted Reproductive Technology and Reproductive Genetics, Shandong University, Jinan, Shandong 250012, China.

[5] Key Laboratory of Reproductive Endocrinology of Ministry of Education, Jinan, Shandong 250001, China.

[6] Shandong Provincial Clinical Research Center for Reproductive Health, Jinan, Shandong 250012, China.

[7] Advanced Medical Research Institute, Shandong University, Jinan, Shandong 250012, China.

† These authors contributed equally to this work as Co-First Authors.

*Correspondence to: zhaibinyuan@sdnu.edu.cn (B.Z.), shunxinwang@sdu.edu.cn (S.W.) or zhangliangran@sdnu.edu.cn (L.Z.)

SUPPLEMENTARY FIGURES

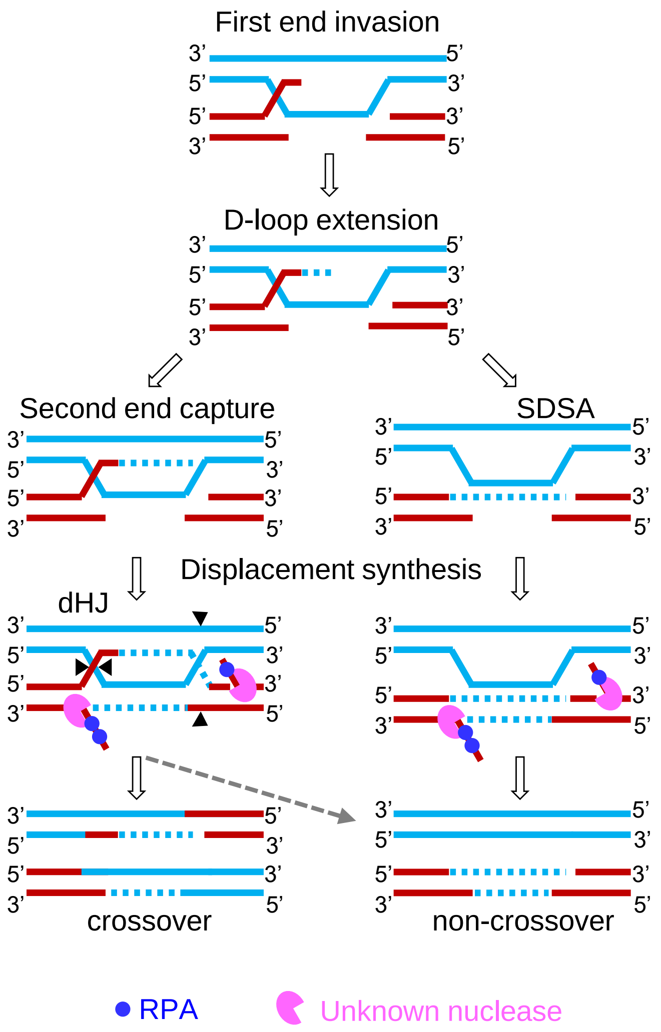

Supplementary Figure 1. Model for meiotic double-strand break (DSB) repair.

One resected DSB end searches and invades the homolog sequence to form the D-loop intermediate. DNA synthesizes from the invasion end. If this structure is stabilized, DNA is continuously synthesized and the second end is captured to form the stable double Holliday junction (dHJ), which is then resolved as a CO. However, if the first end is released from the D-loop structure, the DSB is then repaired as a NCO by the synthesis dependent strand annealing (SDSA) pathway. DNA synthesis in both pathways can produce DNA flaps, which have to be timely removed for DSB repair.

A

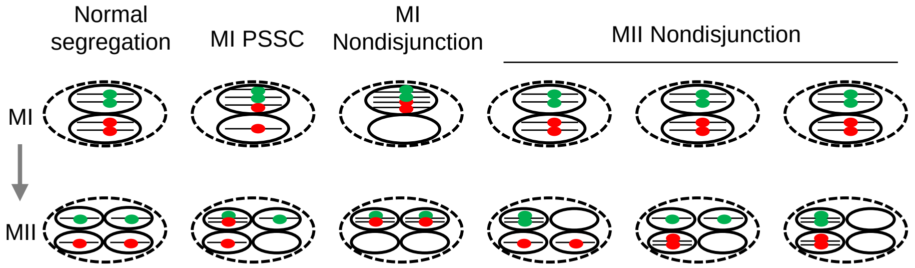

B

Wild type	636/647= 98.3%	1/647 =0.15%	1/647 =0.15%	3/647 =0.46%	6/647 =0.92%	0/647 =0%
dna2-md	409/566= 72.3%	18/566 =3.2%	0/566 =0	49/566 =8.7%	82/566 =14.5%	8/566 =1.4

Supplementary Figure 2. Chromosome mis-segregation assay.

(A) Cartoons to show the patterns of chromosome 9 segregation during meiosis I (MI) and meiosis II (MII). **(B)** The frequency of each type of segregation (corresponding to that in panel A) in WT and *dna2-md*. For the four spores in a tetrad with proper chromosome segregation, two spores are green and the other two are red. The number before the slash indicates the number of tetrads observed in that category and the number after the slash indicates the total number of tetrads examined in that genotype. PSSC, premature separation of sister chromatids.

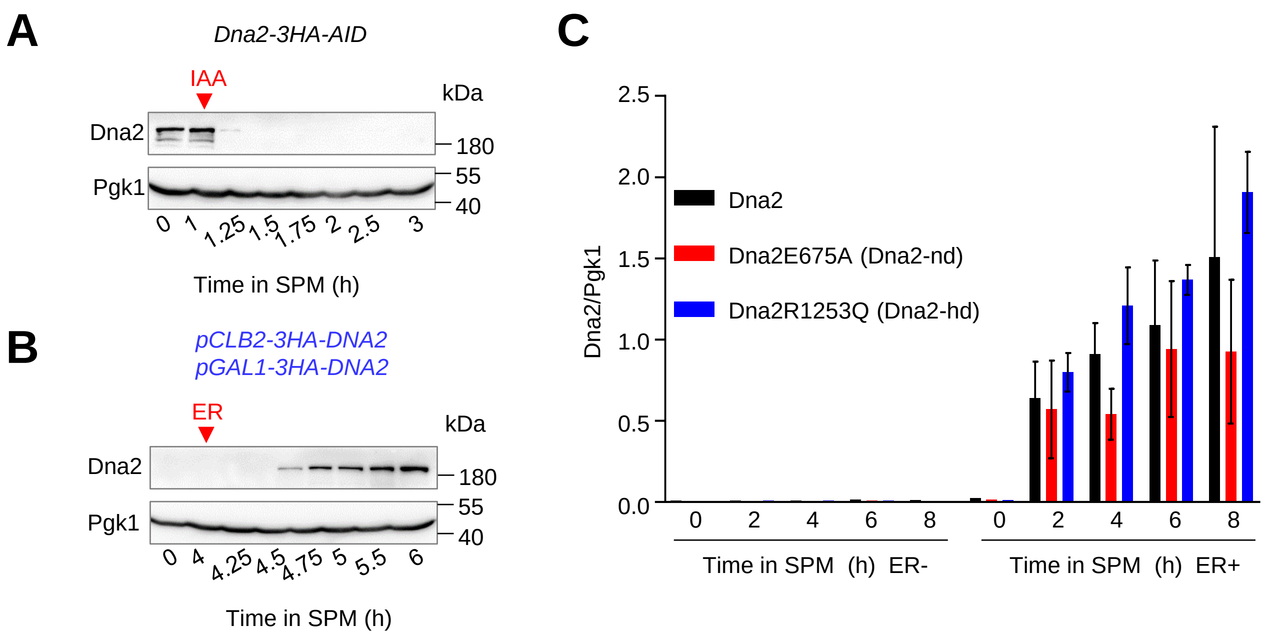

Supplementary Figure 3. Dna2 abundance examined by Western blot.

(A, B) Western blot to show Dna2 abundance. Dna2 degradation was induced by IAA added at 1h in SPM (A). Dna2 expression was induced by β-estradiol added at 4h in SPM (B). **(C)** Quantification of Figure 1O to show the abundance of Dna2, Dna2-hd, and Dna2-nd after induction. Error bar, SEM; n = 3.

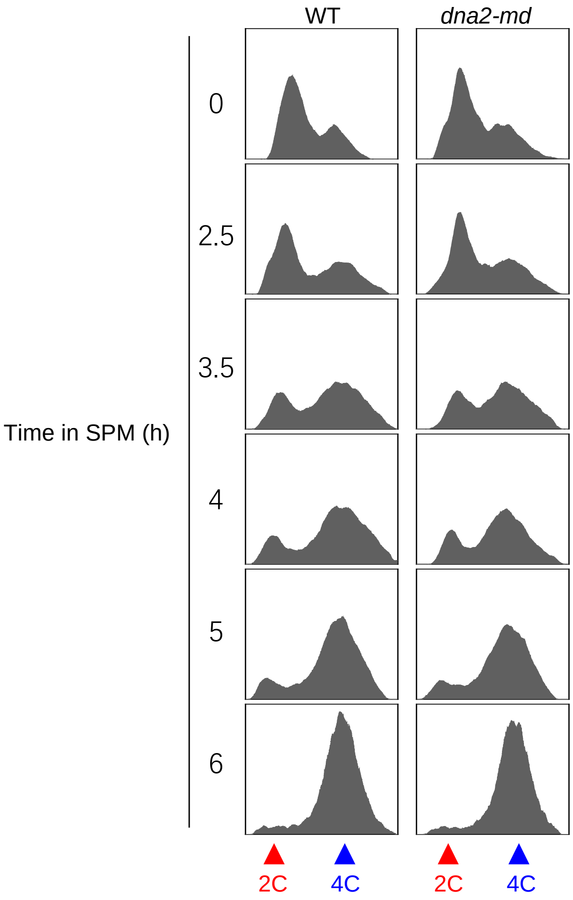

Supplementary Figure 4. The pre-meiotic DNA replication in WT and *dna2-md*.

Cultures were synchronized in SPS medium and transferred to SPM medium to induce meiosis. The pre-meiotic DNA replication was examined by flow cytometry at indicated time points. The peaks for 2C and 4C DNA contents were indicated by red and blue arrowheads, respectively.

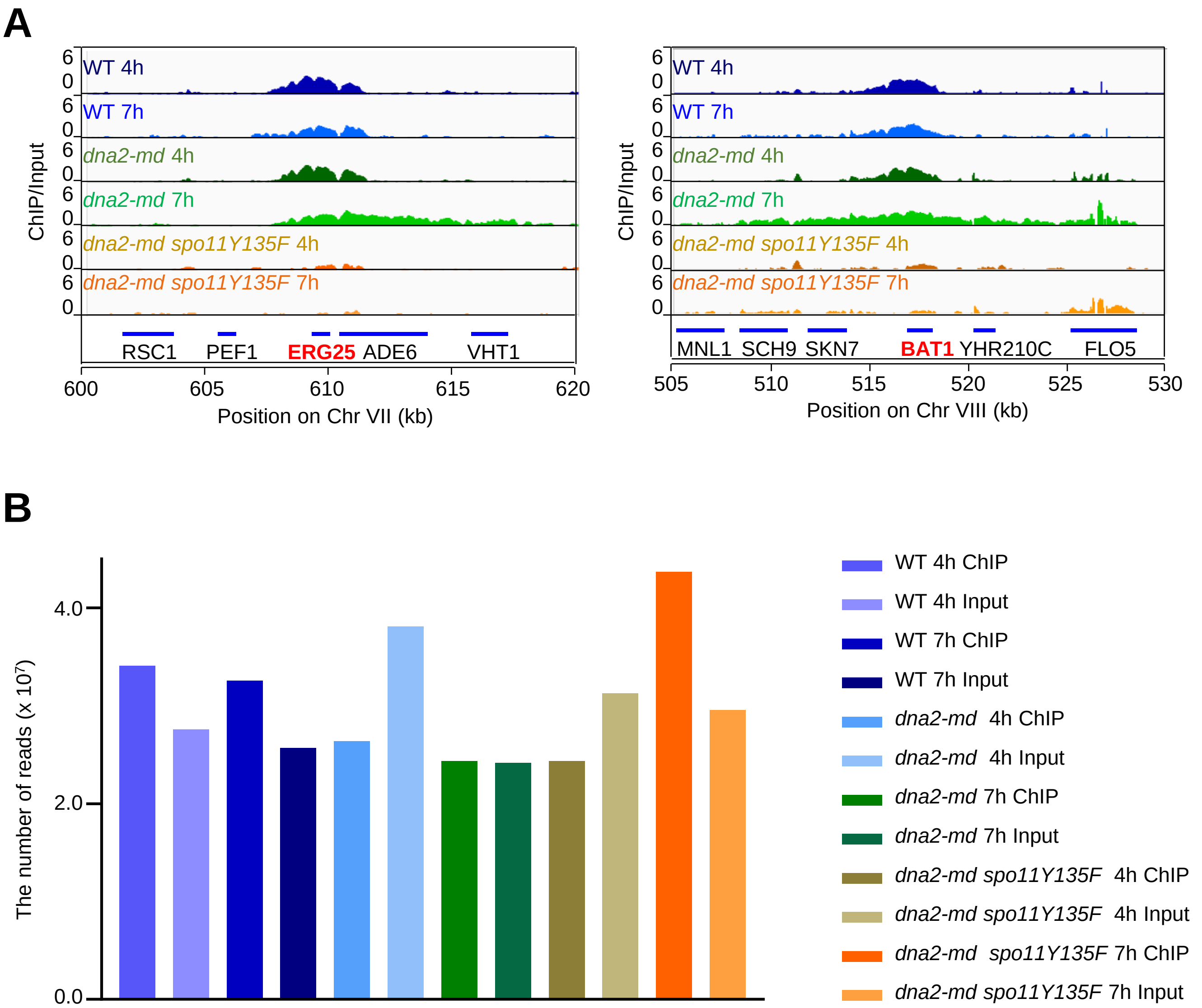

Supplementary Figure 5. RPA extends from DSB centers to the flanking regions in *dna2-md.* **(A)** Snapshots of RPA enrichment at two DSB hotspots, *ERG25* (left panel) and *BAT1* (right panel). The results were visualized using the non-normalized data. **(B)** Total reads number of RPA ChIP-seq in WT, *dna2-md*, and *dna2-md spo11Y135F* at 4h and 7h in SPM, respectively.

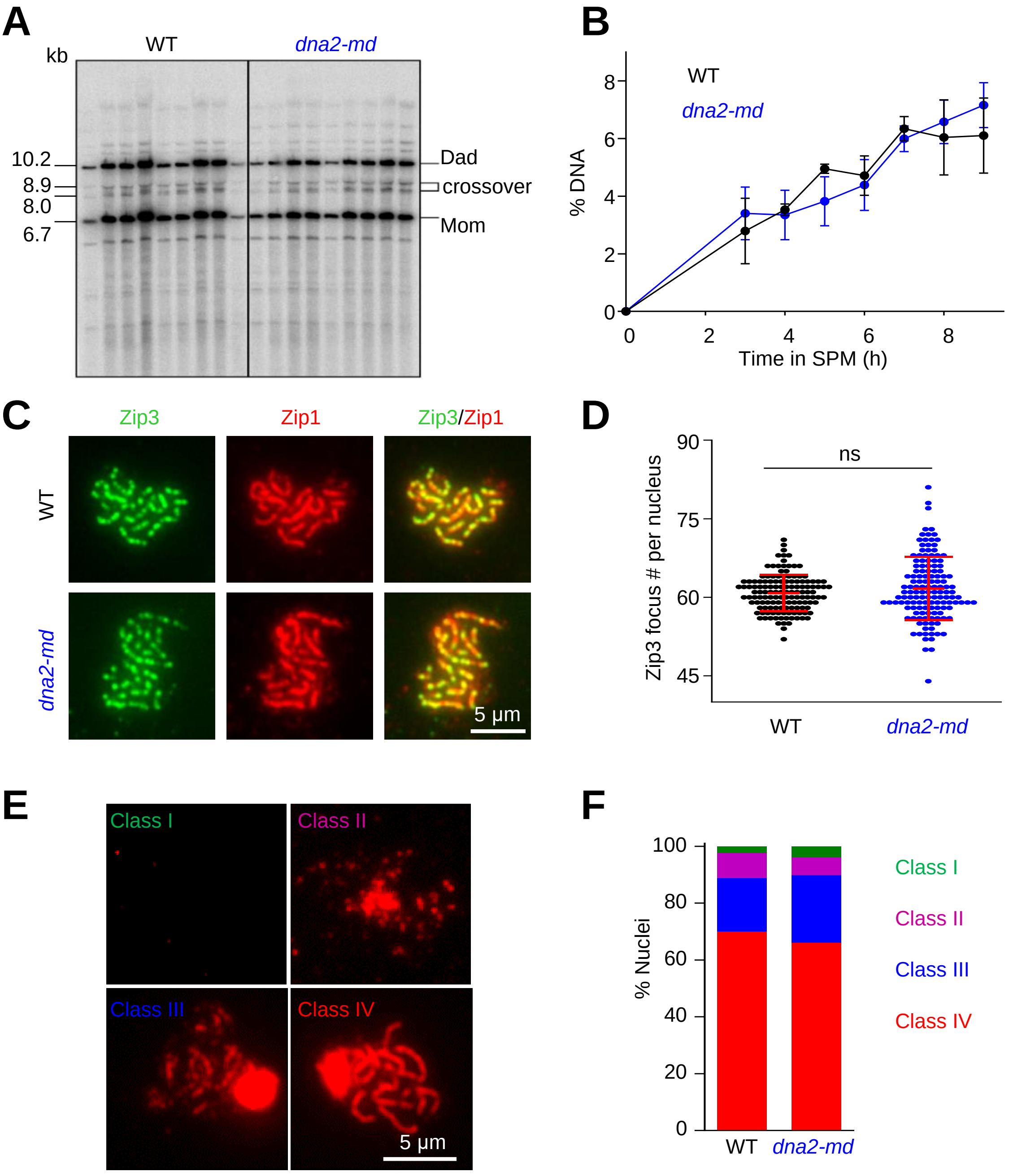

Supplementary Figure 6. Dna2 plays limited roles in CO formation and homolog synapsis.

(A) Representative images to show COs at the *ERG1* hotspot detected on 1D gels. The genomic DNA was digested by SacII. **(B)** Quantification of COs in (A). Error bar, SEM; n = 3. **(C)** Representative images to show CO-associated Zip3 foci in pachytene nuclei in WT and *dna2-md* strains. Scale bar, 5μm. **(D)** Quantification of (C) to show the number of Zip3 foci per nucleus. Totally, 136 WT and 136 *dna2-md* pachytene nuclei were examined. Error bar, SD. Two-tailed Student's t-test; ns (not significant). **(E)** Representative images to show the four classes of Zip1 morphologies in *ndt80Δ* background (10h in SPM). Scale bar, 5μm. **(F)** Quantification of (E) to show the percentages of each class of Zip1 morphology. Totally, 468 WT and 386 *dna2-md* nuclei were examined.

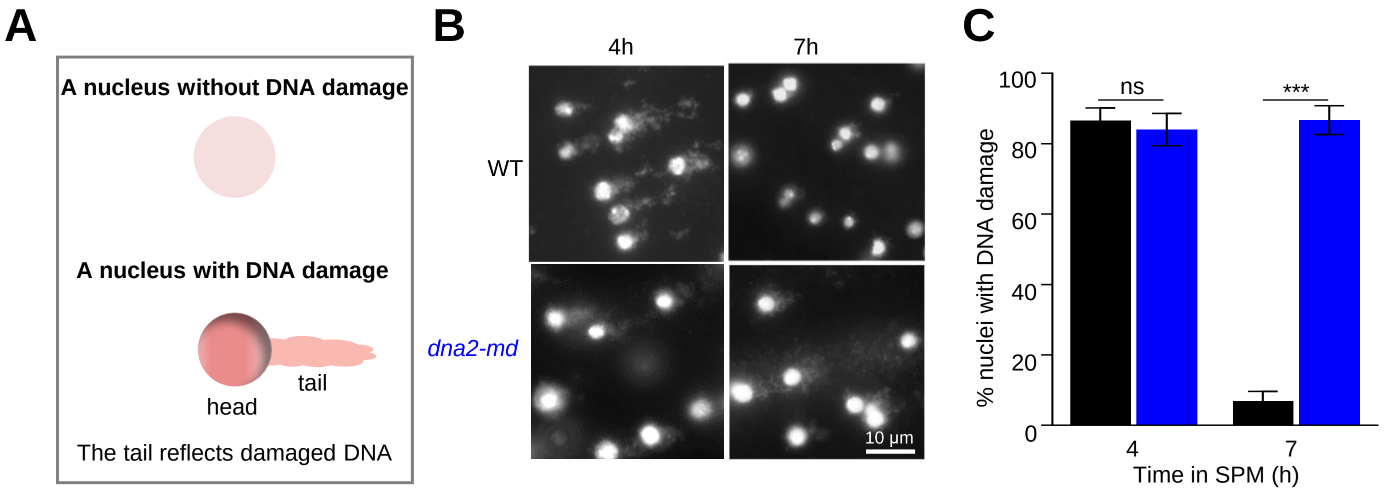

Supplementary Figure 7. The comet assay of DNA damage.

(A) The cartoon shows a nucleus without or only with very little DNA damage (top) and a nucleus with severe DNA damage as indicated by the large tail (bottom). The amount of DNA in the tail indicates the degree of DNA damage. **(B)** Representative images of comet assay in WT and *dna2-md*. Scale bar, 10 μm. **(C)** Quantification of (B) to show the percentage of nuclei with DNA damage. The numbers of nuclei examined were 351 (4h) and 334 (7h) in WT, and 244 (4h) and 263 (7h) in *dna2-md*. Error bar, 95% confidence interval. Two proportion Z-test; ns (not significant), *** ($p < 0.001$).

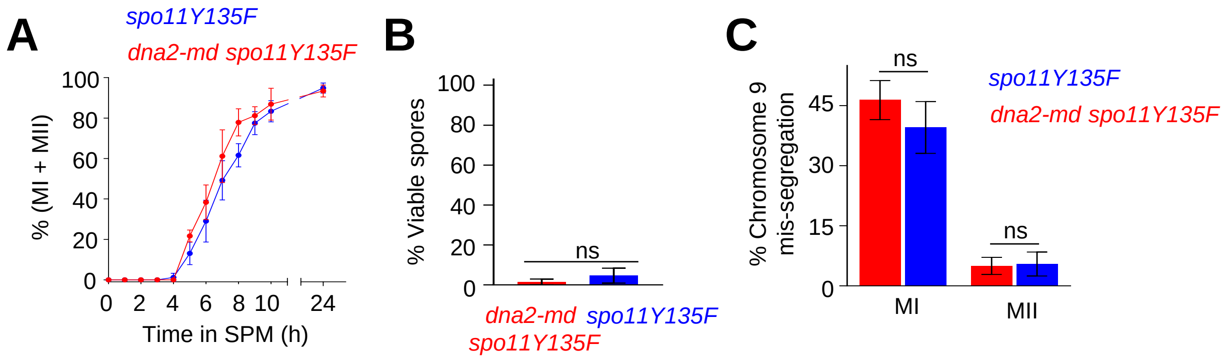

Supplementary Figure 8. Analysis of the phenotypes in *spo11YF* and *dna2-md spo11YF*.

(A) Meiotic nuclear divisions in *dna2-md spo11YF* and *spo11YF*. More than 200 nuclei were examined at each time point in each experiment. Error bar, SEM; n = 3. (**B**) Spore viability. 96 tetrads were analyzed for each strain. Error bar, 95% confidence interval. Two proportion Z-test; ns (not significant). (**C**) The frequency of tetrads with chromosome 9 mis-segregation. Totally, 401 *dna2-md spo11YF* and 220 *spo11YF* tetrads were examined. Error bar, 95% confidence interval. Two proportion Z-test; ns (not significant).

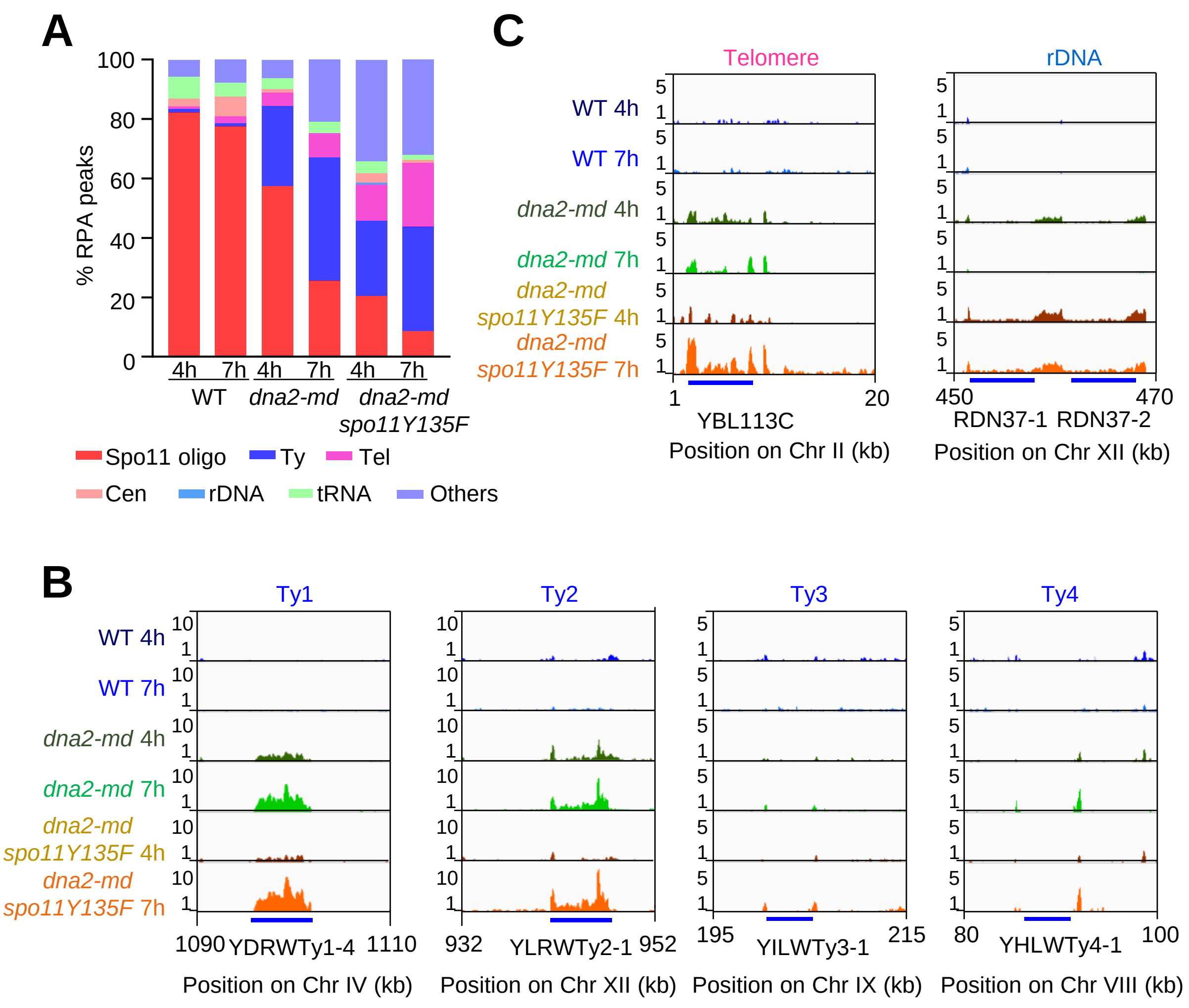

Supplementary Figure 9. Dna2 regulates Spo11-independent RPA accumulation.

(A) The distribution of RPA peaks detected by ChIP-seq in WT and mutants. The numbers of RPA peaks detected were 242 (4h) and 89 (7h) in WT, 332 (4h) and 258 (7h) in *dna2-md*, and 321 (4h) and 302 (7h) in *dna2-md spo11Y135F*. Peaks were called by MACS2. Only RPA peaks ≥ 2-fold enrichment over the background were used for further analysis. **(B)** Snapshots of RPA enrichment at representative Ty1-Ty4 retrotransposons. **(C)** Snapshots of RPA enrichment at telomere and rDNA.

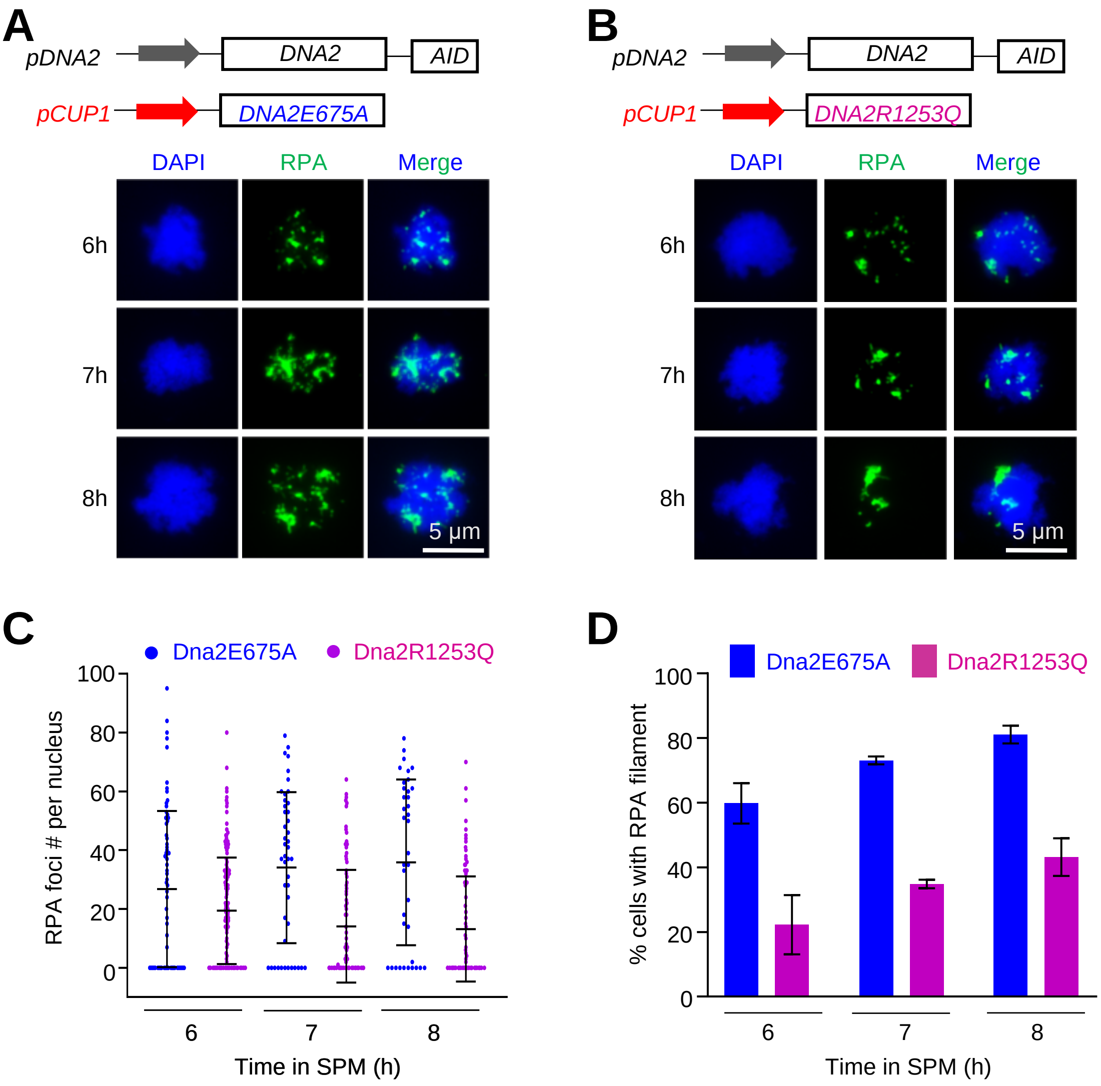

Supplementary Figure 10. Removal of accumulated RPA requires both Dna2 nuclease and helicase activity.

(A, B) Representative images to show RPA accumulation in Dna2 nuclease dead mutant (A) or helicase dead mutant (B). Cartoons on the top indicate strains used. Dna2 degradation was induced with 2 mM IAA and 25 μM Cu^{2+}, meanwhile Dna2E675A (Dna2-nd) or Dna2R1253Q (Dna2-hd) was induced by Cu^{2+}. **(C, D)** Quantification of the numbers of RPA foci (C) and the percentages of cells with RPA filaments (D). Sample size, n = 73, 173, 45, 131, 37, and 97 nuclei, respectively (C). Scale bar, 5 μm. Error bar, SD (C), or the range of two experiments (D).

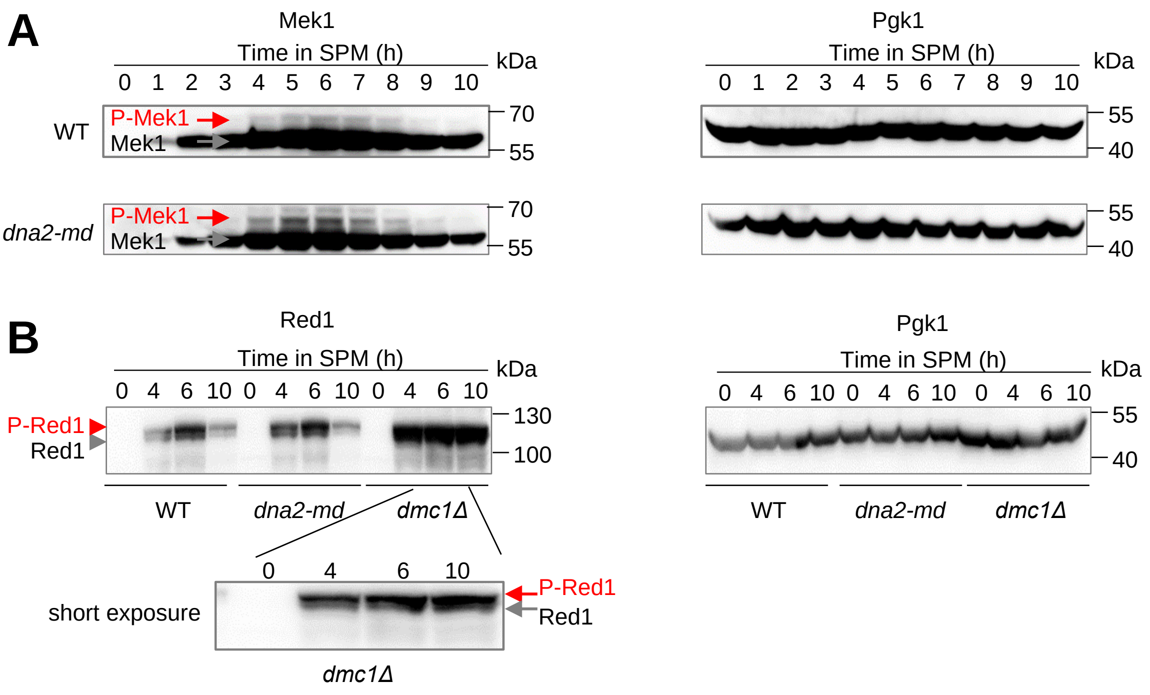

Supplementary Figure 11. Analysis of Mek1 phosphorylation and Red1 phosphorylation in WT and *dna2-md*. **(A)** The dynamics of Mek1 phosphorylation and dephosphorylation during meiosis was examined by Western-blot in WT and *dna2-md*. **(B)** Western blot to show Red1 phosphorylation in WT, *dna2-md*, and *dmc1Δ*.

Table S1. Strains used in this study.

Strain	Genotype*
SWY89	*ho::hisG/", his3/", leu2/",CAN/can, CYH2/cyh2, CEN9:: LEU2, $P^{YKL050c}$RFP/CEN9::HIS3-$P^{YKL050c}$YFP*
NHY4763	*ho/", leu2/", ura3/", ERG1-(Sal1)/ERG1-(SpeI), HIS4::LEU2-(BamHI; +ori)/his4-X::LEU2-(NgoMIV; +ori)-URA*
LZY388	*ho::LYS2/”, ura3/”, leu2::hisG/”, his3::hisG/”, trp1::hisG/”, cdc6::kanMX6::pSCC1:3-HA-CDC6/”*
LZY629	*ho::hisG/", ura3/", leu2/", HIS4::LEU2-(BamHI+ori)/ his4-x::LEU2-(NgoMIV+ori)-URA3, nuc1::hygroB/"*
LZY1035	*ho/", lys2? leu2::hisG/", ura3/", dmc1::KanMx4/", ZIP3-GFP::URA3/", MEC1-myc18::LEU2/”*
LZY819	*ho::hisG/", ura3/",leu2/", ZIP3-13MYC::Hygromycin B/", URA3::CYC1p-LacI-GFP/", SCP1::LacO-LEU2/"*
LZY3011	*ho::hisG/", ura3/", leu2/", REC8-3HA::URA3/", ZIP3-13MYC::Hygromycin B/", ndt80::LEU2/"*
LZY3436	as LZY819, except *pCLB2-DNA2::KanMx4/”*
LZY3525	as SWY89, except *pCLB2-DNA2::KanMx4/”*
LZY3528	as LZY629, except *pCLB2-DNA2::KanMx4/”*
LZY3531	as LZY4166, except *pCLB2-DNA2::KanMx4/”*
LZY3614	as LZY819, except *DNA2-3HA::NAT/”*
LZY4166	as LZY629, except *rad50KI81::URA3/”*
LZY4902	*ho::LYS2/”, lys2/”, ura3/”, leu2::hisG/”, REC8-3HA::URA3/”*
LZY4903	as LZY4902, except *pCLB2-DNA2::KanMx4/”*
LZY4904	*ho/hisG/”, lys2/”,leu2/”, ura3/”, DNA2-AID$^{71-114}$-6HA::NatMX4/”, pCUP1-OsTIR1::URA3/”*
LZY4905	*ho::hisG/ho::LYS2, leu2/”, lys/”, ura3::PGPD-GAL4(848)-ER-URA3/ura3, REC8-3HA::URA3/”, pGAL1-DNA2::NatMx4/ pCLB2-DNA2::KanMX*
LZY4906	*ho::hisG/ ho::LYS2, leu2/”, lys/”, ura3::PGPD-GAL4(848)-ER-URA3/”, REC8-3HA::URA3/”, pCLB2-DNA2::KanMX/”, his3::pGAL1-DNA2::NAT/”*
LZY4907	*ho::hisG/ ho::LYS2, leu2/”, lys/”, ura3::PGPD-GAL4(848)-ER-URA3/”, REC8-3HA::URA3/”, pCLB2-DNA2::KanMX/”, his3::pGAL1-DNA2E675A::NAT/”*
LZY4908	*ho::hisG/ ho::LYS2, leu2/”, lys/”, ura3::PGPD-GAL4(848)-ER-URA3/”, REC8-3HA::URA3/”, pCLB2-DNA2::KanMX/”, his3::pGAL1-DNA2R1253Q::NatMX4/”*
LZY4909	as LZY4903, except *spo11(Y135F)::HphMx4/”*
LZY4910	as LZY4902, except *spo11(Y135F)::HphMx4/”*
LZY4911	as LZY4903, except *ZIP3-13myc::Hygromycin B/”*
LZY4912	as LZY4902, except *pCLB2-PIF1::NAT/”*
LZY4913	as LZY4903, except *pCLB2-PIF1::NAT/”*
LZY4914	as LZY4903, except *ndt80::LEU2/”*
LZY4915	as LZY4906, except *ndt80::LEU2/”*
LZY4916	as LZY4906, except *ndt80::LEU2/pGAL1-NDT80::NAT*
LZY4917	as LZY4902, except *mlh2::NAT/”*

LZY4918	as LZY4903, except *mlh2::NAT/"*
LZY5003	as LZY388, except *pCLB2-DNA2::KanMx4/"*
LZY5005	as NHY4763, except *pCLB2-DNA2::KanMx4/"*
LZY5204	*ho::LYS2/", lys2/", ura3/", leu2::hisG/", arg4-bgl/ arg4-nsp, his4B-LEU2/ his4X::LEU2(Bam)-URA3, GPD-HSV-TK-URA3/", hENT1-AUR1-C/"*
LZY5205	as LZY5204, except *pCLB2-DNA2::KanMx4/"*
LZY5206	*ho::hisG/", lys2/", ura3::hisG/", leu2::hisG/", ade2/", Mek1-3HA::URA3/"*
LZY5207	as LZY5206, except *pCLB2-DNA2::KanMx4/"*
LZY5208	as LZY4913, except *mlh2::NAT/"*
LZY5209	as LZY4904, except *his3::pCUP1-DNA2E675A::NAT/"*
LZY5210	as LZY4904, except *his3::pCUP1-DNA2R1253Q::NAT/"*
LZY5211	as LZY3525, except *spo11(Y135F)::HphMx4/"*
LZY5212	as SWY89, except *spo11(Y135F)::HphMx4/"*

***** All strains are derived from the SK1 background.
